# Developing an m5C regulator–mediated RNA methylation modification signature to predict prognosis and immunotherapy efficacy in rectal cancer

**DOI:** 10.3389/fimmu.2023.1054700

**Published:** 2023-02-22

**Authors:** Rixin Zhang, Wenqiang Gan, Jinbao Zong, Yufang Hou, Mingxuan Zhou, Zheng Yan, Tiegang Li, Silin Lv, Zifan Zeng, Weiqi Wang, Fang Zhang, Min Yang

**Affiliations:** ^1^ State Key Laboratory of Bioactive Substances and Function of Natural Medicine, Institute of Materia Medica, Chinese Academy of Medical Sciences and Peking Union Medical College, Beijing, China; ^2^ Clinical Laboratory, The Affiliated Hospital of Qingdao University, Qingdao, China; ^3^ Qingdao Hospital of Traditional Chinese Medicine, The Affiliated Qingdao Hiser Hospital of Qingdao University, Qingdao, China

**Keywords:** rectal cancer, prognosis, tumor immune microenvironment, immunotherapy, m5C RNA methylation regulator

## Abstract

**Background:**

Currently, a very small number of patients with colorectal cancer (CRC) respond to immune checkpoint inhibitor (ICI) treatment. Therefore, there is an urgent need to investigate effective biomarkers to determine the responsiveness to ICI treatment. Recently, aberrant 5-methylcytosine (m^5^C) RNA modification has emerged as a key player in the pathogenesis of cancer. Thus, we aimed to explore the predictive signature based on m^5^C regulator–related genes for characterizing the immune landscapes and predicting the prognosis and response to therapies.

**Methods:**

The Cancer Genome Atlas (TCGA) cohort was used as the training set, while GEO data sets, real-time quantitative PCR (RT-qPCR) analysis from paired frozen tissues, and immunohistochemistry (IHC) data from tissue microarray (TMA) were used for validation. We constructed a novel signature based on three m^5^C regulator–related genes in patients with rectal adenocarcinoma (READ) using a least absolute shrinkage and selection operator (LASSO)-Cox regression and unsupervised consensus clustering analyses. Additionally, we correlated the three-gene signature risk model with the tumor immune microenvironment, immunotherapy efficiency, and potential applicable drugs.

**Results:**

The m^5^C methylation–based signature was an independent prognostic factor, where low-risk patients showed a stronger immunoreactivity phenotype and a superior response to ICI therapy. Conversely, the high-risk patients had enriched pathways of cancer hallmarks and presented immune-suppressive state, which demonstrated that they are more insensitive to immunotherapy. Additionally, the signature markedly correlated with drug susceptibility.

**Conclusions:**

We developed a reliable m^5^C regulator–based risk model to predict the prognosis, clarify the molecular and tumor microenvironment status, and identify patients who would benefit from immunotherapy or chemotherapy. Our study could provide vital guidance to improve prognostic stratification and optimize personalized therapeutic strategies for patients with rectal cancer.

## Introduction

By blocking programmed cell death 1/programmed cell death ligand 1 (PD1/PDL1) axis, immune checkpoint inhibitors (ICIs) have introduced a new era of antitumor therapy that could elicit durable responses and significantly improve survival in several tumors (1, 2). However, the contexture and organization of the immune environment can be highly heterogeneous among tumors, even within the same cancer type, leading to a complex crosstalk within the tumor immune microenvironment (TIME) (3). The overall status of tumor-infiltrating lymphocytes (TILs) in TIME closely correlates with the efficacy of immunotherapy. According to the immune cell status in TIME, tumor immune infiltration pattern could be broadly classified into “hot tumor” (indicating presence of CD8+ and CD4+ T cells accompanied by high expression of immune checkpoint molecules) and “cold tumor” (representing the deficiency of immune cells within the tumor parenchyma) (4, 5). The former has a potential antitumor efficacy, while the latter barely benefits from the ICI therapy (6). At present, patients with deficient mismatch repair (dMMR)/microsatellite instability-high (MSI-H) have more immune cell infiltration accompanied by high tumor mutational burden (TMB), while microsatellite stable (MSS)/microsatellite instability-low (MSI-L) patients have low abundance of TILs and low TMB (7, 8). Moreover, according to the KEYNOTE-016 study, 62% of colorectal cancer (CRC) patients with MSI-H phenotype achieve an objective response, while patients with MSS/MSS-L tumors cannot achieve objective response, indicating a better efficacy of immunotherapy in patients with dMMR/MSI-H tumors (9). Nonetheless, dMMR/MSI-H tumors account for only 15% of all patients with CRC (7, 10). Therefore, establishing effective predictive biomarkers is essential for the improvement of immunotherapeutic strategy.

RNA modification plays an important role in the regulation of gene expression. More than 150 RNA modifications containing N6-methyladenosine (m^6^A), 5-methylcytosine (m^5^C), and N1-methiadenosine (m^1^A) have been investigated (11, 12). Among these modifications, m^5^C is one of the most intensively researched epigenetic modifications, and overall, 95391 m^5^C sites in the human genome have been identified (13). The m^5^C methylation landscape is regulated by a dynamic process that integrates methyltransferases (“writer”), binding proteins (“readers”), and demethylases (“erasers”) (14, 15). Although m^5^C is widely recognized for its essential function as an epigenetic marker for DNA, research into its functional roles in RNA is beginning to emerge. It has been shown that a vast majority of azactidine (5-AZA), widely used to treat hematologic malignancies, is incorporated into RNA instead of DNA of treated tumor cells (16). Therefore, the potential use of m^5^C RNA modification as a novel therapeutic target for various types of cancers is a current topic of research.

RNA methylation impacts the efficacy of tumor immunotherapy by modulating immune activity in a range of tumors (17). Recently, several studies have uncovered the close relationship between TIME-infiltrating immune cells and m^5^C RNA methylation. Pan et al. found that NOP2/Sun RNA methyltransferase 4 (NSUN4) and NOP2/Sun RNA methyltransferase 3 (NSUN3) were closely related to the infiltration by six major immune cells that could regulate TIME in lung squamous cell carcinoma (18). Gao et al. showed that m^5^C RNA modification patterns could predict and affect TIME in oral squamous cell carcinoma (19). Despite these facts, the relationship between RNA methylation and tumor immunotherapy is still in its infancy. In the current study, we integrated multiple data sets and developed a novel signature based on the expression of m^5^C RNA methylation regulators, which could be used to evaluate risk status and predict prognosis of patients with rectal adenocarcinoma. Furthermore, we comprehensively explored the correlations between the m^5^C RNA methylation regulator–based signature having immune characteristics, mutational burden, and immunotherapeutic and chemotherapeutic sensitivity in READ (rectal adenocarcinoma) patients. Our results suggested that the established signature based on m^5^c RNA methylation regulators could be used as a robust biomarker to predict the clinical prognosis and therapeutic effect among patients with rectal cancer.

## Materials and methods

### Acquisition and processing of data sets

The RNA-sequencing transcriptome data (TPM value) and corresponding clinical annotation were retrieved from The Cancer Genome Atlas (TCGA) database (http://gdc-portal.nci.nih.gov/). After patients without survival information were excluded, a total of 434 colon adenocarcinoma (COAD) and 157 READ samples were integrated for further analysis. The validation data set was retrieved from the Gene Expression Omnibus (GEO) database (https://www.ncbi.nlm.nih.gov/geo/) under the accession number GSE87211 (n=190) (20) and GSE133057 (n=17) (21). The copy number variations (CNV) of READ used in our research were retrieved from the UCSC Xena browser (http://xena.ucsc.edu/), where genes with CNV values smaller than −0.3 were categorized as a “loss,” while CNV values larger than 0.3 were categorized as a “gain.” The messages of simple nucleotide variations (SNV) were retrieved from the TCGA database, R package *maftools* was used to analyze the level 4 mutation data, and the *mafCompare* function of *maftools* was used to identify the differentially mutated genes (DMGs) (22). The neoantigens and mutation loads for READ were accessed from The Cancer Immunome Atlas (https://tcia.at/) database (23). Information on CMS subtyping calls and sample annotations were retrieved from the Colorectal Cancer Subtyping Consortium Synapse (24). The STRING database can predict the functional links between proteins based on a variety of algorithms. The genes with the highest confidence scores were identified as the functional partners of specific genes (25). The Gene_DE module of Tumor Immune Estimation Resource (TIMER, cistrome.shinyapps.io/timer) can be utilized to examine the mRNA expression profiles between the tumor tissues and the normal tissues (26). We used the Human Protein Atlas (HPA) database to analyze the protein expression levels of candidate genes in tumor tissues and corresponding normal tissues (27).

### Construction of gene signature and survival analysis

The least absolute shrinkage and selection operator (LASSO) model is a linear regression method applying L1-regularization, which could accurately contract some regression coefficients to zero to achieve sparseness and feature selection (28). The LASSO model was generated through R package *glmnet*. At the penalty coefficient (λmin = 0.036), the optimal risk model was established based on three m^5^C regulatory genes. Next, the R package *survival* was used to calculate the risk scores for rectal cancer samples. The following formula was used:


Risk score=e∧(constant+∑i coefficient(mRNAi)×expression(mRNAi))

Patients from the TCGA training cohort were separated into a high-risk and a low-risk group according to the median value of the calculated risk score. Patients from the GEO validation data set were grouped based on the optimal cutoff decided by *cutp* function of the R package *survMisc*. The Kaplan–Meier method was employed to compare the survival probability between the two risk subgroups.

### Functional enrichment analysis

Differentially expressed genes (DEGs) between the subgroups were identified by R package *limma*. Metascape (http://metascape.org), a web tool comprising Gene Ontology (GO) and Kyoto Encyclopedia of Genes and Genomes (KEGG) analysis (29), was used to identify the terms across different ontology sources enriched based on the screened DEGs. A GOCircle plot was depicted to show the enriched terms by R package Goplot (30). To further investigate pathways enriched in specific subgroups, we performed Gene Set Variation Analysis (GSVA) by R package *GSVA*. GSVA is a gene set enrichment method that estimates variation of pathway activity over a sample population in an unsupervised manner (31). The gene set of “c5.go.v7.4.symbols” was downloaded from MSigDB database, and gene markers of epithelial–mesenchymal transition (EMT) including EMT1, EMT2, and EMT3; angiogenesis; pan-fibroblast TGFβ; and type I IFN response were obtained from previous studies for GSVA analysis (32, 33).

### Immune cell infiltration analysis

A total of 28 immune cell types were collected for GSVA analysis (23). A web server TIMER, integrating multiple algorithms (TIMER, Cell-type Identification By Estimating Relative Subsets Of RNA Transcripts [CIBERSORT], European Prospective Investigation into Cancer and Nutrition [EPIC]) was used to estimate the abundances of immune cell types based on the gene expression profiles (26, 34, 35). The ratios between immune-stimulatory signatures and immune-inhibitory signatures (CD8+/CD4+ regulatory T cells, pro-/anti-inflammatory cytokines, and M1/M2 macrophages) were also compared between the subgroups based on the average expression levels of the marker genes (36). The immune system–related genes were obtained from previous studies (23, 37–39). The Pearson correlation was calculated and then depicted by R package *corrplot*.

### Prediction of the efficacy of immunotherapy and chemotherapy

A web platform named Tumor Immune Dysfunction and Exclusion (TIDE, http://tide.dfci.harvard.edu) was used to evaluate the anti-PD1 and anti-CTLA4 immunotherapeutic response based on the gene expression profiles of the TCGA-READ cohort (40). To validate the correlation between immunotherapeutic efficacy and three genes–based risk model, another data set was retrieved, which included 348 patients with metastatic urothelial cancer who were treated with an anti-PD-L1 agent (32). The R package *oncoPredict* can be used to discover drug sensitivity *in vitro* and *in vivo* contexts (41). The half-maximal inhibitory concentration (IC50) was calculated to predict the chemotherapeutic response in READ patients. The Cancer Therapeutics Response Portal (CTRP, https://portals.broadinstitute.org/ctrp/) (42) and Profiling Relative Inhibition Simultaneously in Mixtures (PRISM, https://depmap.org/portal/prism/) (43) were both developed to access the associations between drug sensitivity and gene expression. The calcPhenotype function of R package oncoPredict was used to calculate the AUC (Area Under Curve) value of each drug based on the CTRP and PRISM databases. Lower AUC value indicates higher sensitivity to therapeutic drugs.

### Consensus clustering analysis

To further validate the reliability of discriminating patients with rectal cancer into two subgroups based on the three m^5^C regulatory genes, DEGs were identified through R package *limma* between the low- and high-risk groups. Furthermore, univariate Cox regression analysis was carried out by R package *survival* to filter prognostic genes on the basis of DEGs. Ultimately, unsupervised clustering analysis was conducted by using R package *ConsensuClusterPlus*, which was repeated 1,000 times to identify different risk gene clusters (44).

### Tissue microarray–based immunohistochemistry validation

From Superbiotek in Shanghai, China (#REC1601), we acquired a TMA of 80 paired rectal cancers and corresponding normal tissues. Surgical samples from the patients were taken between May 2008 and December 2012 through operations. The patients’ median survival duration was 81.5 months, ranging from 14 to 130 months. For every case, clinicopathological information including overall survival time, survival status, age, gender, tumor size, pathological T, N, and M stage, and grade was accessible. Based on this commercial TMA, we conducted a retrospective analysis.

For immunohistochemistry (IHC) process, the TMA slides were deparaffinized, rehydrated, and incubated by 3% hydrogen peroxide to block the endogenous peroxidase activity for 10 min at room temperature. Antigens were restored by boiling in a pressure cooker containing sodium citrate buffer for 90 s. The slides were incubated in bovine serum albumin (BSA) for 30 min to reduce nonspecific background. Then, they were incubated with rabbit monoclonal NSUN4 antibody (HPA028489, Sigma), NSUN7 antibody (HPA020653, Sigma), and DNMT1 antibody (HPA002694, Sigma) at 4°C overnight. Next, secondary antibody was incubated with the slides for 1 h at 37°C. Finally, the slides were developed in 3, 3’-diaminobenzidine (DAB) and stained with hematoxylin.

The slides were assessed digitally with the APERIO ScanScope (Leica Biosystems, Germany) and the APERIO ImageScope (Leica Biosystems, Germany) using the positive pixel counting algorithm. The IHC staining results were interpreted by both the intensity of staining and the staining positive area. Each sample was assigned a score according to the intensity of the staining (0 = no staining; 1 = weak staining; 2 = moderate staining; and 3 = strong staining) and the proportion of stained cells (0 = 0%; 1 = 1%–25%; 2 = 25%–50%; 3 = 50%–75%; 4 = 75%–100%). The final score was calculated as the staining intensity multiplying positive area score, ranging from 0 to 12. The IHC results of TMA-rectal cancer were independently reviewed by two experienced pathologists who were blinded to the clinical parameters.

### Real-time quantitative PCR validation

For the RT-qPCR experiment, tissue samples from 26 rectal cancer patients and matched nearby normal tissue samples (proximity to the cancer larger than 5 cm) were collected at the Affiliated Hospital of Qingdao University. The inclusion requirements were as follows (1): a pathological analysis and imaging-based diagnosis of rectal cancer; (2) radical resection; (3) available information on clinicopathological indexes, such as tumor size, pathological stage, and pathological TNM; (4) pathological TNM in accordance with the 8^th^ edition of the American Joint Committee on Cancer; and (5) lack of a prior history of other malignancies. Patients with recurrent rectal cancer and nonprimary malignancies as well as those who had had neoadjuvant chemotherapy and/or radiation prior to surgery were disqualified. All of the included patients gave their informed permission. The Affiliated Hospital of Qingdao University’s Research Ethics Committee approved the study, and it was completed in conformity with the 1964 Helsinki Declaration and its later amendments.

Total RNA was extracted using RNeasy kit (Beyotime, Shanghai, China, R0027) in accordance with the manufacturer´s instructions. Then, total RNA (1 µg) was quantified, followed by reverse-transcription by the SuperScript II reverse transcriptase (Takara, Japan, RR047). Quantitative PCR analysis was operated using SYBR Green Mix (Takara, Japan, RR820) with ABI 7900 HT Real-Time PCR system. The primer sequences are listed below: NSUN4, 5’-CCAAACCCTGGCAAAAGGTG-3’, 5’- GCGTGCCGGTCATAGAAGAA-3’; NSUN7, 5’-CCAGATCATTTGAGCAGTCTTATT-3’, 5’- GGTTCTCTACTTCTTGAACTTCTGA-3’; DNMT1, 5’-ATCCGAGGAGGGCTACCTG-3’, 5’- ACTTCTTGCTTGGTTCCCGT-3’; GAPDH, 5’-CTGACTTCAACAGCGACACC-3’, 5’-TGAGCTTGACAAAGTGGTCGT-3’. mRNA levels were determined relatively according to the expression of GAPDH.

### Statistical analysis

The t-test or Wilcoxon test was adopted for comparisons of two groups, and one-way ANOVA or Kruskal–Wallis test was adopted for comparisons of three or more groups. The choice of t-test vs. Wilcoxon test, or one-way ANOVA vs. Kruskal–Wallis test, was based on the normality of the variables. Chi-squared tests were used to analyze the distribution of variables among different subgroups. Multivariate Cox regression analysis was carried out by R package *survival*. Receiver operating characteristic (ROC) analysis was used to evaluate the predictive power of the established model. We constructed nomograms to predict survival probability using R package *rms*. *P* value less than 0.05 was recognized as significant in this research.

## Results

### Construction of m^5^C RNA methylation regulator–based signature for READ patients

**Figure 1:**
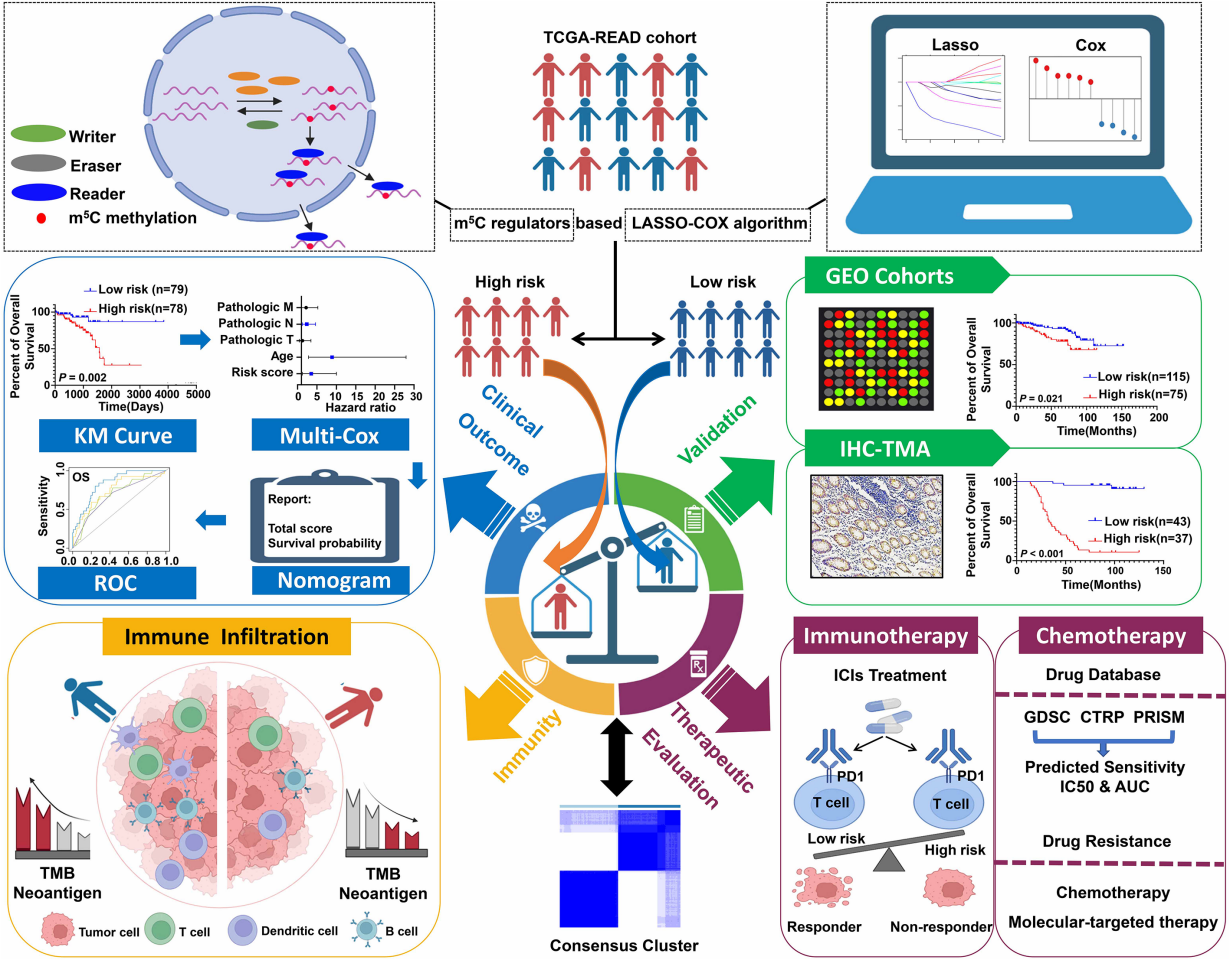
Schematic diagram of the study design.

**Figure 2:**
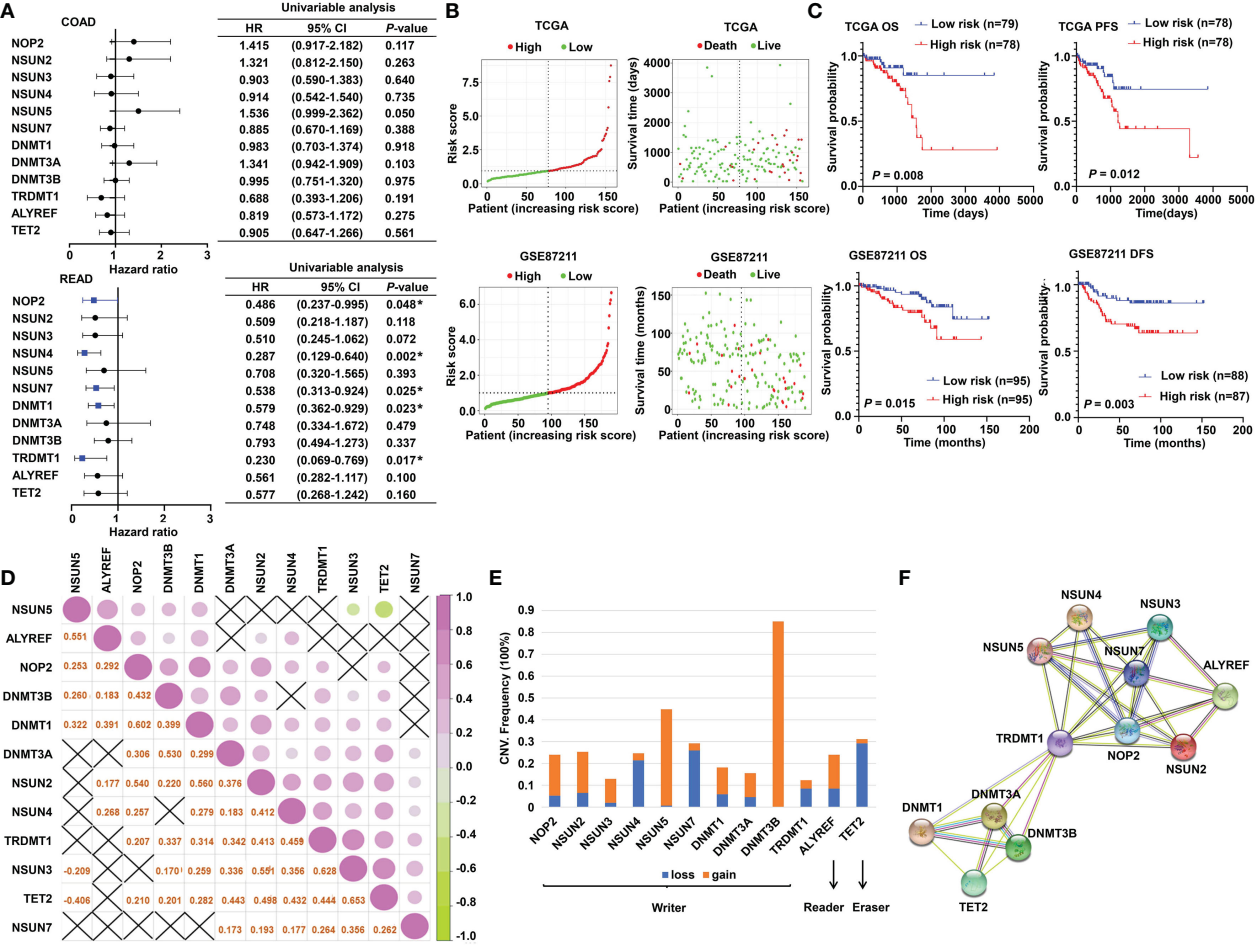
The prognostic value of m^5^C RNA methylation regulators. **(A)** Forest plot of the prognostic ability of the m^5^C regulator genes in COAD and READ separately. **(B)** The risk score distribution and patient survival status are shown in ranked dot and scattered plots based on the expression of m^5^C regulator genes. **(C)** The Kaplan–Meier curves for OS, PFS, and DFS between the high-risk and the low-risk groups in READ from the TCGA and GSE87211 cohorts. **(D)** Pearson correlation among the m^5^C regulators in READ patients. **(E)** The CNV variation frequency of the m^5^C regulators in the TCGA cohort. The orange rectangle = the amplification frequency; the blue rectangle = the deletion frequency. **(F)** The PPI network depicted for m^5^C regulators. CI, confidence interval; DFS, disease-free survival; HR, hazard ratio; OS, overall survival; PFS, progression-free survival. *P < 0.05.

The schematic diagram summarizes the study design of the current research (**Figure 1**). m^5^C RNA modification regulators (NOP2 nucleolar protein [NOP2], NOP2/Sun RNA methyltransferase [NSUN]2, NSUN3, NSUN4, NSUN5, NSUN7, DNA methyltransferase [DNMT]1, DNMT3A, DNMT3B, tRNA aspartic acid methyltransferase 1 [TRDMT1], Aly/REF export factor [ALYREF], and tet methylcytosine dioxygenase 2 [TET2]) were integrated in this research based on the previously published articles (40, 45). To explore the function of these regulators, univariate Cox analyses were conducted for COAD and READ separately. Interestingly, we found that the m^5^C modification regulators mainly played their roles in READ contrasting with COAD; specifically, NOP2, NSUN4, NSUN7, DNMT1, and TRDMT1 functioned as protective factors for patients of READ (**Figure 2A**). Therefore, in the following research, we focused mainly on the functions of the m^5^C RNA modification regulators related to READ. Owing to the observation of the prognostic value of the m^5^C regulators, we explored the overall prognostic impact of these regulators on READ. We built a prognostic model based on the mRNA expression value of total m^5^C regulators multiplying hazard coefficients to predict the survival events of READ patients. Next, the patients were classified into two groups (**Figure 2B**). As expected, the high-risk group presented a worse survival rate than the low-risk group, which was observed both in TCGA and in GSE87211 data sets (**Figure 2C**). Correlations among the mRNA expression levels of the m^5^C modification regulators were analyzed by Pearson correlation analysis. The results exhibited a whole trend of positive correlation among m5C regulatory genes (**Figure 2D**), and protein–protein interactions were calculated using String data sets (**Figure 2F**), which demonstrated that the m5C regulators could play an integrated role in impacting the prognosis of patients with READ. The CNV events were also examined by retrieving the mutation data from the TCGA-READ cohort. NSUN4, NSUN7, and TET2 had a tendency to a loss of copy number, while the remaining regulators often showed copy number gain events. Specifically, DNMT3B showed the most frequent CNV events, followed by NSUN5 (**Figure 2E**), implying that m^5^C regulators play an important role in the process of m^5^C modification in READ. These results indicated the potential potency of the m^5^C regulators as prognostic biomarkers for READ patients.

**Figure 3:**
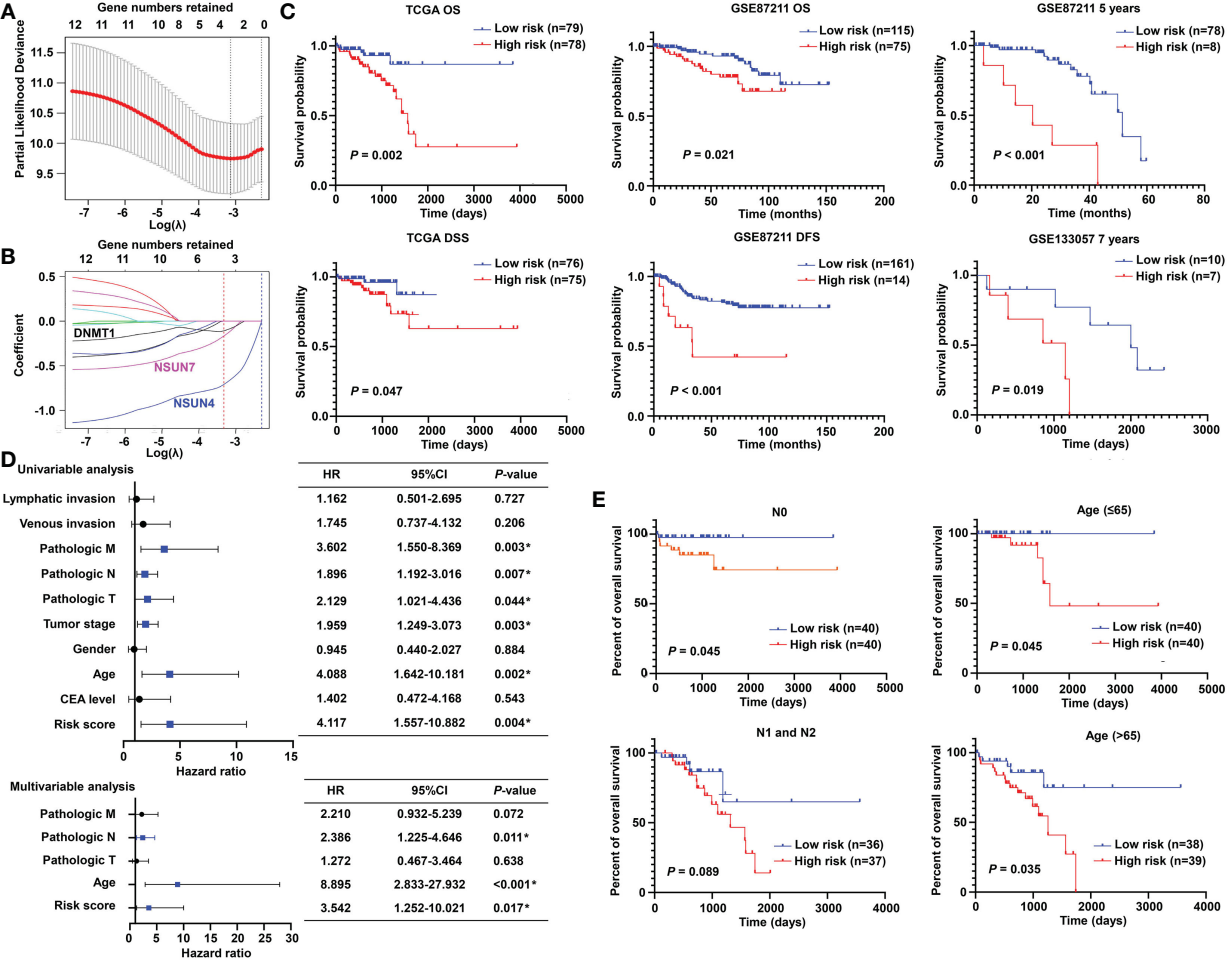
Prognostic significance of the m^5^C methylation-based signature in READ patients. **(A)** The process of LASSO regression based on the TCGA cohort and the identification of “lambda” for best selection of gene signature. **(B)** The curves indicate the tracks of single genes; the red dot line represents the target lambda. The blue track refers to NSUN4, pink track refers to NSUN7, and black line is DNMT1. **(C)** The Kaplan–Meier curves for OS and DSS between two categories in READ from the TCGA data set; the Kaplan–Meier curves for OS, 5-year survival based on the GSE87211 data set; the Kaplan–Meier curves for 7-year survival based on the GSE133057 data set. **(D)** The univariate and multivariate Cox analysis integrating risk score and clinicopathological indexes based on the TCGA cohort. **(E)** The prognostic ability of the risk score in distinguishing the overall survival status for READ patients with or without lymph node metastasis. The prognostic ability of the risk score in differentiating the overall survival status in patients with age less than 65 years or those with 65 years or more. CI, confidence interval; DFS, disease-free survival; DSS, disease-specific survival; HR, hazard ratio; OS, overall survival. *P < 0.05.

To promote the clinical application, LASSO-penalized Cox analysis was performed to enhance the forecast accuracy and explainability of the statistical model. In the current model, the optimal penalty coefficient (λ = 0.036, log λ = −3.33) was identified with the minimum criterion (**Figure 3A**). In **Figure 3B**, each curve indicates the track of a single gene, and the red dot represents the target lambda. We can see that three genes (DNMT1, NSUN4, and NSUN7) were retained after the shrinking process. Then, the produced three prognostic indicators were employed to predict clinical results.

### Prognostic significance of the m^5^C methylation–based signature in READ patients

To confirm the effectiveness of the established model, we carried out Kaplan–Meier survival analyses. We found a superior survival status in the low-risk group compared with the corresponding high-risk group in both the TCGA dataset and two GEO cohorts, illustrating that the built model could significantly predict the prognosis of READ patients (**Figure 3C**). Similar processes were applied to the samples of COAD patients, and no factor was retained after LASSO analysis (**Figure S1A**). The three factors identified in the READ patients were repurposed for COAD samples; as expected, the survival curves of the two groups were highly crossed (**Figure S1B**). To further explore the relationship of the prognostic risk model of the three m^5^C regulators and clinical features in READ, univariate and multivariate Cox regression analyses were conducted. To facilitate the understanding of the patients’ clinical and genetic background, a table including basic information about the low- and high-risk groups is displayed in **Table S1**. The results of Cox regression analysis revealed that the risk score was an independent prognostic factor for READ, unrelating to clinicopathological parameters, such as pathologic N and age (**Figure 3D**). We further investigated whether the risk score could further subdivide the pathological N and age parameters. The results showed that the established risk score further distinguished the risk pattern in subgroups differentiated by age, successfully stratified the patients in the N0 pathological stage, and exhibited a tendency to differentiate patients in the N1 pathological stage due to small sample size (**Figure 3E**). To visualize the expression pattern of m^5^C regulators, a heat map was depicted. To our expectation, the majority of the methylation regulators displayed a significant high expression module in the low-risk group (**Figure S2**), which is reasonable due to their protective ability in READ. Thus, this powerful and accurate model symbolized a potential clinical parameter for patients with READ.

### Construction and validation of a nomogram combined with clinical parameters

**Figure 4:**
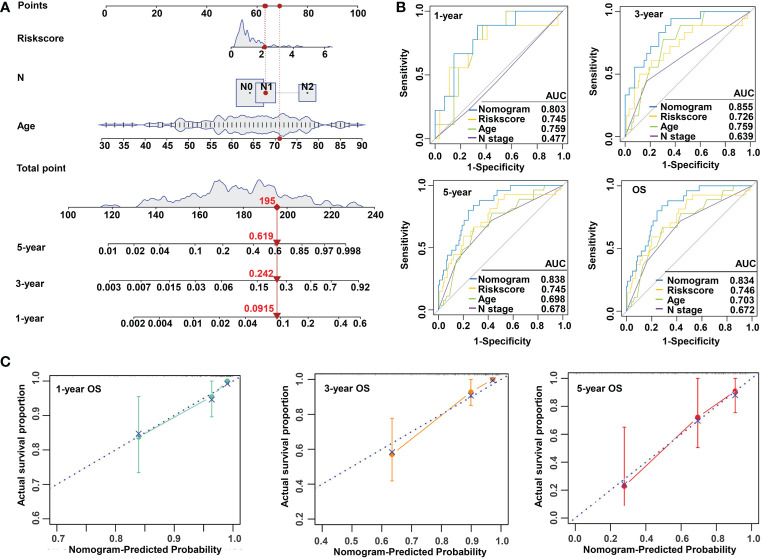
Construction and validation of a risk model based on m^5^C methylation regulators. **(A)** The predictive nomogram integrating the risk score and clinicopathological parameters for 1-, 3-, and 5-year OS in READ patients from the TCGA cohorts. **(B)** The ROC for nomogram and independent clinical parameters for 1-, 3-, and 5-year OS based on the TCGA cohort in READ patients. **(C)** The calibration curve depicted for 1-, 3-, and 5-year nomogram in TCGA. OS, overall survival.

To make the m^5^C regulator–based risk signature more clinically adapted and available, a prognostic nomogram was depicted integrating the risk factors and independent identified parameters of READ. The aim was to establish a quantitative analytic algorithm that could be put into practice for survival prediction. In the current case, the pathologic N, age, and risk score were integrated to calculate the corresponding score, which could be used as an index for matching the one-, three-, and five-year death probabilities (**Figure 4A**). To reinforce the superior capability of the established nomogram, the ROC analyses were used to compare the prognostic accuracy and specificity. The results indicated that the nomogram was superior to other independent clinical factors for predicting the overall survival (OS) of READ patients in the TCGA cohort (AUC of one-year OS = 0.803; AUC of three-year OS = 0.855; AUC of five-year OS = 0.838; AUC of overall survival = 0.834; **Figure 4B**). The calibration curve was drawn to confirm the consistency between the nomogram-predicted and the actual probability. The calibration curves were close to the optimal performance in the one-, three-, and five-year nomogram (**Figure 4C**), indicating the accuracy of the constructed nomogram. These results implied that the three-gene signature was capable and reliable to make prediction for READ patients.

### Functional enrichment analysis of m^5^C methylation–based signature between low- and high-risk READ patients

**Figure 5:**
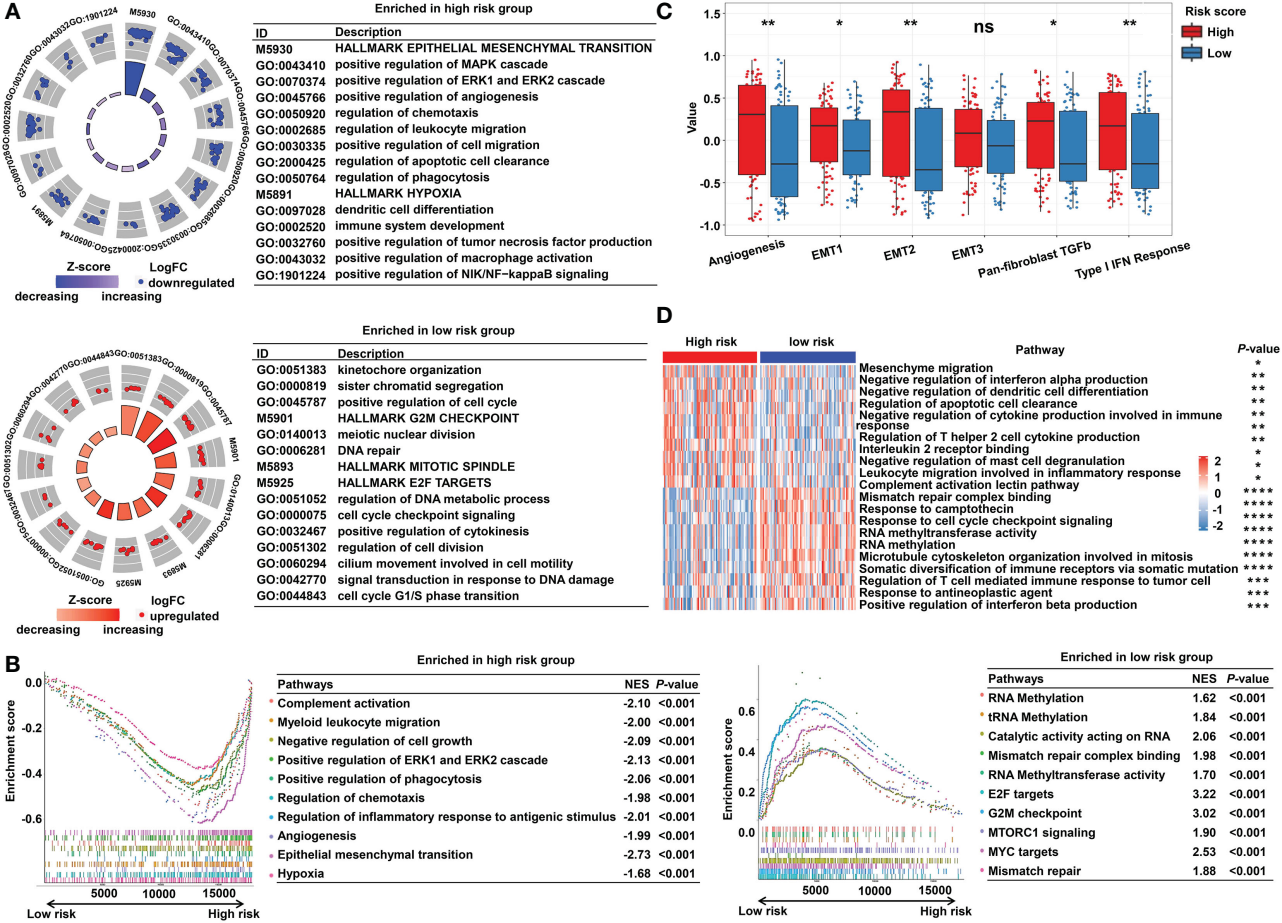
Functional enrichment analysis of the m^5^C methylation-based signature between low- and high-risk READ patients. **(A)** The enriched pathways including GO and HALLMARK terms are displayed by GOcircle plots. The red and blue dots represent the genes upregulated in the low-risk and high-risk categories separately. **(B)** GSEA enrichment plots for the two subgroups in the TCGA cohort. **(C)** The GSVA analysis for hallmarks of cancer in the TCGA cohort. **(D)** The heat map drawn for GSVA analysis based on GO terms. DEGs, differentially expressed genes. ****P < 0.0001; ***P < 0.001; **P < 0.01; *P < 0.05; ns, not significant.

To explore the underlying molecular mechanisms of the m^5^C-based signature, GO, GSEA, and GSVA analyses were performed. DEGs were identified using the limma algorithm, and the result is displayed as a volcano plot (**Figure S3**). Next, the screened DEGs were put into the GO analysis. The GO pathway enrichment analysis revealed that the most significantly changed pathways in the high-risk subgroup were mainly related to cancer and immune-targeted processes, such as epithelial–mesenchymal transition, angiogenesis, hypoxia, regulation of leukocyte migration, and regulation of macrophage activation; however, cell cycle–related pathways, including G2M checkpoints, sister chromatid segregation, and signal transduction in response to DNA damage, were mainly converged in the low-risk group (**Figure 5A**). The GSEA analysis confirmed these findings and showed some extent of overlap with the GO analysis results (**Figure 5B**). In order to clarify the specific roles of these pathways according to the risk categories, a series of related gene sets were collected to further carry out the GSVA analysis. Importantly, the GSVA results revealed that the process of angiogenesis, EMT, and pan-fibroblast TGFβ were consistently upregulated in the high-risk category (**Figure 5C**). Meanwhile, the GSVA analysis indicated that many biological functions in the high-risk group primarily correlated with inflammatory responses and carcinogenic reactions, while in the low-risk group, RNA methylation process and drug response were significantly enriched (**Figure 5D**). These features gave the hint that cancer–immunity interaction is the potential mechanism of the m^5^C-based risk signature, and the efficacy of the established model was further validated by the above results.

### The immune characteristics of the m^5^C regulator–based signature in READ

**Figure 6:**
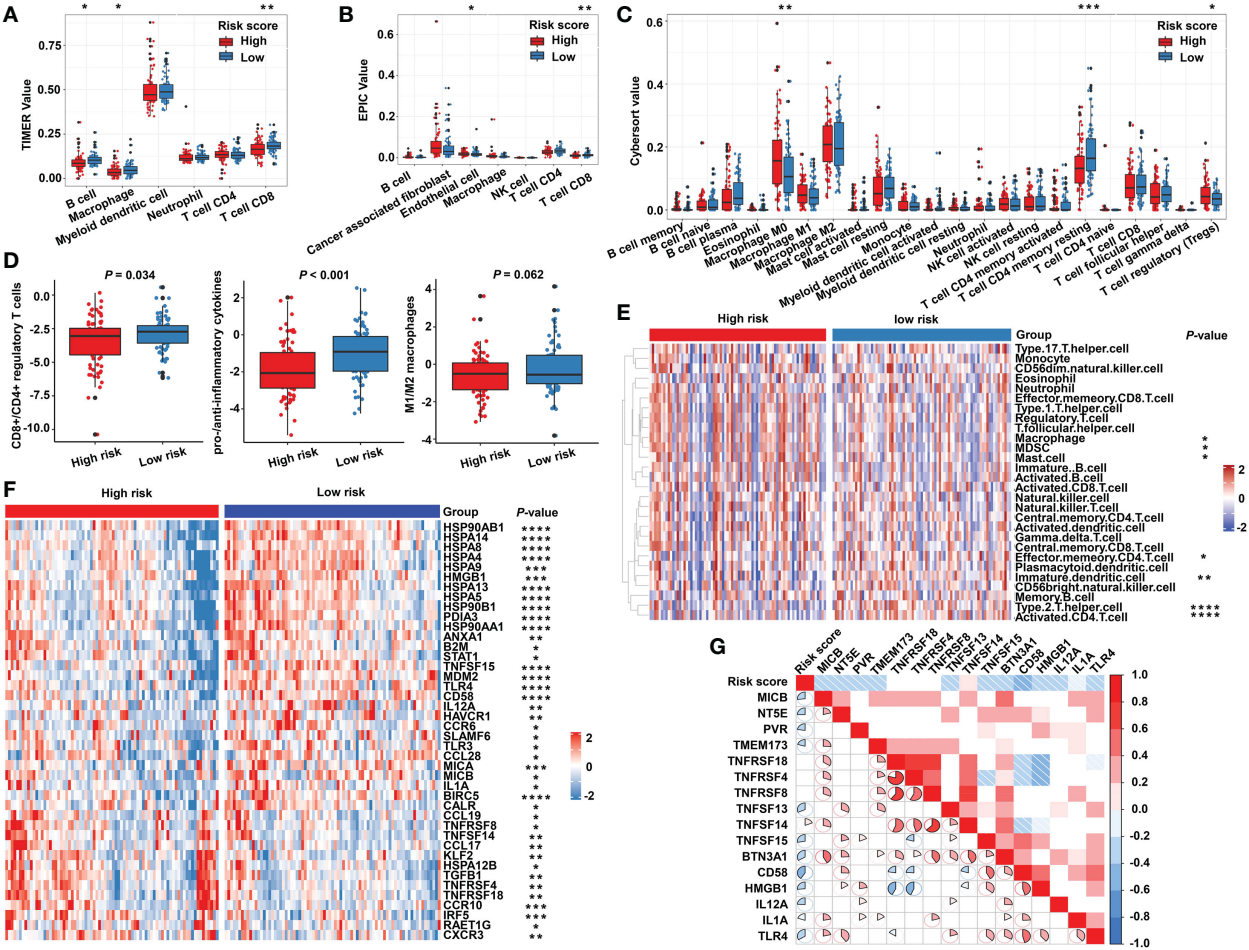
The immune characteristics of the m^5^C regulator–based signature in READ. The deconvolution algorithms of TIMER **(A)**, EPIC **(B)**, and CIBERSORT **(C)**, which were applied to estimate the immune infiltration status between the high- and low-risk groups. **(D)** The ratios of CD8+ T cells to CD4+ regulatory T cells, pro- to anti-inflammatory cytokines, and M1 to M2 macrophages in the TCGA dataset. **(E)** GSVA analysis based on GO terms for the high- and low-risk groups. **(F)** The heat map depicts the expression of positive genes collected from cancer–immunity cycle based on the TCGA cohort. **(G)** Pearson correlation among immune stimulators was conducted and is shown in a correlation heat map. Correlations with P value > 0.05 are blank. ****P < 0.0001; ***P < 0.001; **P < 0.01; *P < 0.05.

Due to the close relationship between the built model and immune process, the detailed connection between the risk signature and immune cell abundance was studied. The GSVA and deconvolution algorithms including CYBERSORT, TIMER, and EPIC were used to evaluate the extent of infiltrating immune cells. CD4+ T cells, B cells, CD8+ T cells, dendritic cells, and T helper cells exhibited higher expression in the low-risk category; meanwhile, the abundance of myeloid-derived suppressor cells (MDSC) and regulatory T cells (Tregs) was elevated in the high-risk category compared with the low-risk group (**Figures 6A–C, E**). Furthermore, additional investigations were conducted to substantiate the above findings. The ratio between the immune stimulatory signatures (including CD8+ T cells, proinflammatory cytokines, and M1 macrophages) and the immune inhibitory signatures (integrating CD4+ regulatory T cells, anti-inflammatory cytokines, and M2 macrophages) was significantly increased in the low-risk category (**Figure 6D**), which was consistent with the above results, indicating an immune-inhibiting environment in the high-risk group and a proinflammatory status in the low-risk group. We collected the signatures of cancer–immunity cycle and immune stimulators. The heat maps showed that the majority of genes exhibited higher expression in the low-risk group (**Figure 6F**) and the established risk score correlated negatively with the expression of most of the immune stimulators (**Figure 6G**). According to the obtained evidence, the low-risk group belongs to activated immune microenvironment, while the high-risk group shows a suppressed immune phenotype.

### The mutational landscape for the m^5^C regulator–based signature in READ

**Figure 7:**
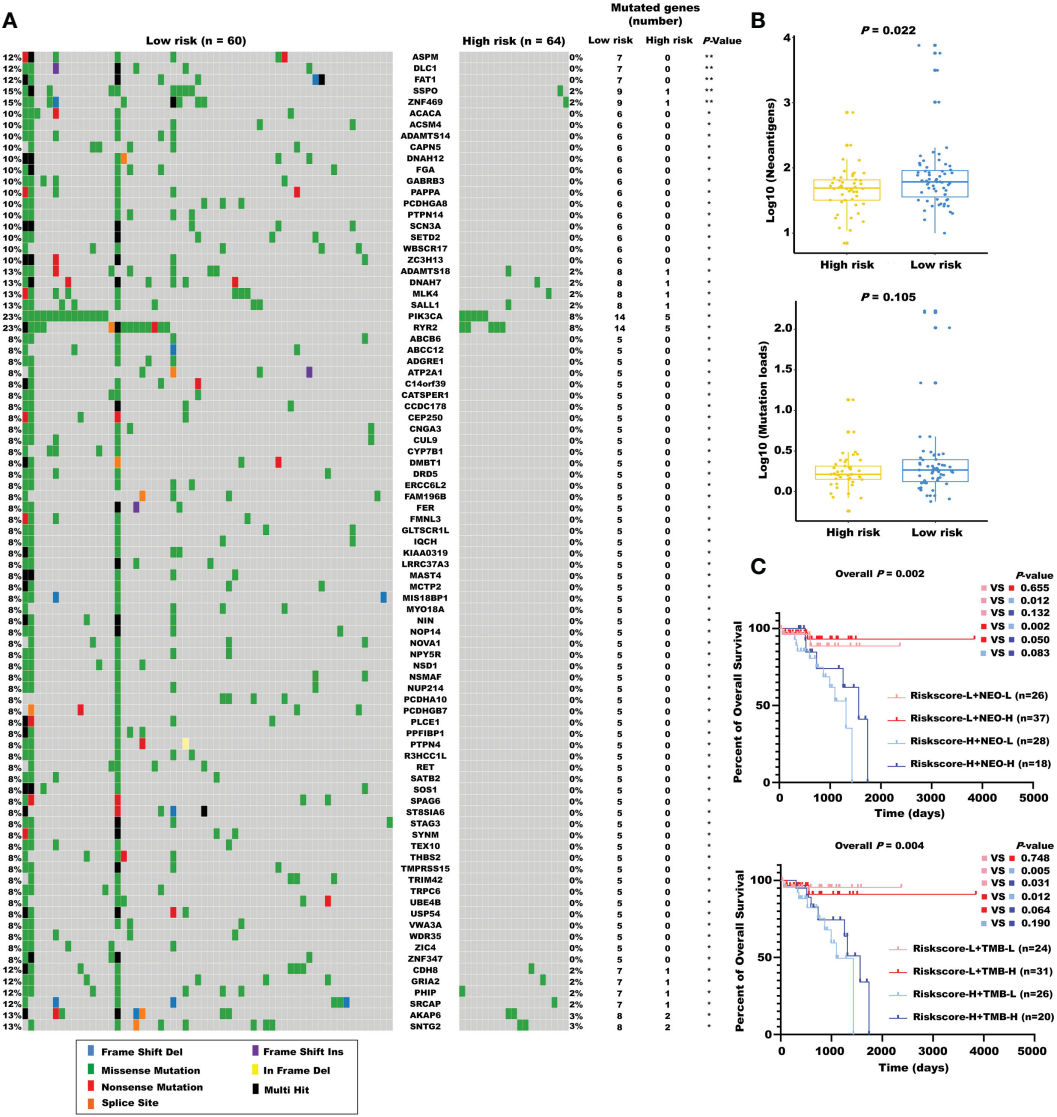
The mutational landscape for the m^5^C regulator–based signature in READ. **(A)** The waterfall plot of differentiated somatic mutation features between the high- and low-risk groups using the TCGA-READ data set. **(B)** The neoantigens and mutation loads between the two subgroups are displayed. **(C)** Survival analyses for READ patients stratified by both the risk score and neoantigen burden or mutation loads using Kaplan–Meier curves. NEO, neoantigen burden; H, high; L, low. **P < 0.01; *P < 0.05.

Considering the evidence that hot tumor is more sensitive to immune therapy, we hypothesized that the low-risk group of our established model might be more readily responsive to immune therapies than the high-risk group. Previous studies have revealed that high somatic mutation and neoantigens represent a higher possibility to response. Thus, we investigated the differences in mutation status between the two groups. First, we identified the top 10 mutated genes in rectal cancer using the maftools R package (**Figure S4A**), and these genes were subsequently compared between the two subgroups. A significantly higher mutational rate of RYR2 was observed in the low-risk group, while the other genes showed no statistical differences (**Figure S4B**). Then, we used the *mafCompare* function to identify the DMGs. Interestingly, we found overall higher mutational rates in the low-risk group (**Figure 7A**), indicating that the built model did not affect the frequently mutated genes but exerted a cumulative effect of low-frequency mutations. We also found that the low-risk group was accompanied by more neoantigens. However, TMB only exhibited an elevated tendency (**Figure 7B**). Moreover, we combined the m5C-based model with neoantigens and TMB and found that neoantigens and TMB cannot effectively distinguish the survival status in patients with rectal cancer (riskscore-L + NEO-L vs. riskscore-L + NEO-H, *P* = 0.655; riskscore-L + TMB-L vs. riskscore-L + TMB-H, *P* = 0.748), although possessing high neoantigen levels showed a tendency of better overall survival compared with possessing low neoantigen levels (riskscore-H + NEO-L vs. riskscore-H + NEO-H, *P* = 0.083). The constructed risk score showed significant efficacy in stratifying patients with a same status of neoantigens and TMB (riskscore-L + NEO-L vs. riskscore-H + NEO-L, *P* = 0.012; riskscore-L + NEO-H vs. riskscore-H + NEO-H, *P* = 0.050; riskscore-L + TMB-L vs. riskscore-H + TMB-L, p = 0.005), confirming the superiority of this model over current biomarkers. In addition, riskscore-L + TMB-H vs. riskscore-H + TMB-H (*P* = 0.064) showed a strong tendency without significant difference. We also found that combining risk score with neoantigens (riskscore-L + NEO-H vs. riskscore-H + NEO-L, *P* = 0.002) could achieve a higher efficiency for predicting the prognosis of patients with rectal cancer (**Figure 7C**).

### Prediction of immunotherapeutic response for distinct subgroups in READ

**Figure 8:**
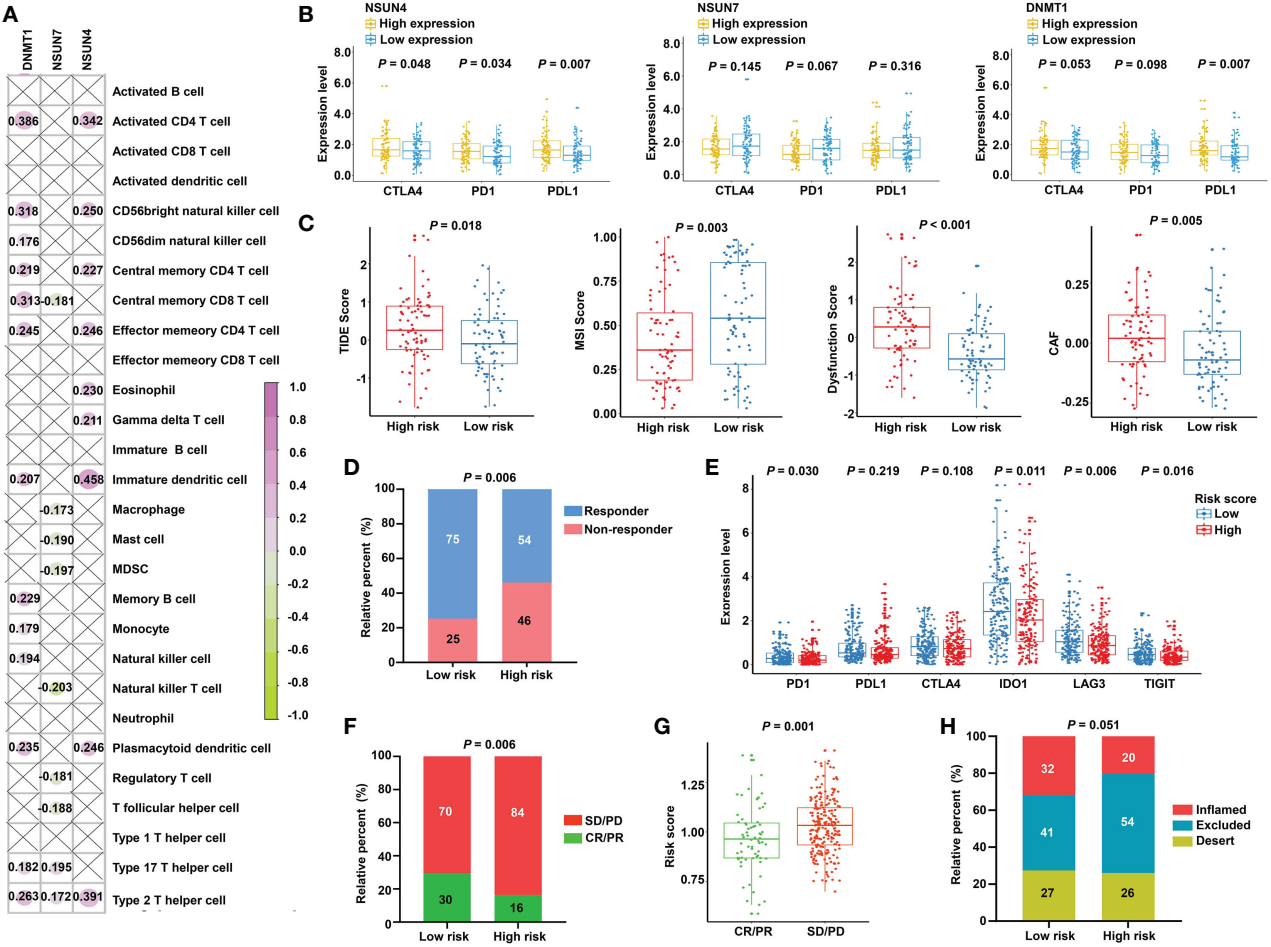
Prediction of immunotherapeutic response for distinct subgroups in READ. **(A)** Pearson correlation between three signature factors and 28 types of immune cells is illustrated by a correlation heat map. Correlations with P value > 0.05 are marked by a cross. **(B)** The differences in the three signature factors between distinct subgroups classified by the expression level of three immune checkpoints, including CTLA4, PD1, and PDL1. **(C)** The distribution of TIDE score, MSI score, T-cell dysfunction score, and abundance of CAF between the low- and high-risk categories. **(D)** The proportion of READ patients with response to ICI therapy in the high- and low-risk groups based on TIDE prediction. **(E)** The differential analysis for immune checkpoints between the two categories in IMvigor210 cohort. **(F)** The proportion of patients with response to PD-L1 treatment in the high- and low-risk groups based on IMvigor210 cohort. **(G)** Distribution of the risk score between CR/PR and SD/PD groups. **(H)** The proportion of immune phenotype in the high- and low-risk groups. CR, complete response; PD, progressive disease; PR, partial response; SD, stable disease.

The obtained findings promoted us to further examine the relationship between the m^5^C-based signature and immunotherapy. First, we compared the expression of the immune checkpoints in the two subgroups. No significant differences were found, as shown in **Figure S5**. Next, we investigated the relationship between model factors and immune infiltration cells. Interestingly, we found that DNMT1 and NSUN4 were moderately positively correlated with CD4+ T cells, natural killer cells, dendritic cells, and T helper cells; meanwhile, NSUN7 was weakly negatively correlated with MDSC and Tregs (**Figure 8A**), substantiating the close connection between the risk model based on the above three m5C regulatory genes and the tumor immune microenvironment. Next, we investigated the relationship between model factors and immune checkpoints. Higher expression of NSUN4 was accompanied by a higher expression of immune checkpoints, and patients with high DNMT1 expression showed a trend of elevated expression of immune checkpoints. However, low expression of NSUN7 was associated with only weakly elevated immune checkpoint expression (**Figure 8B**). A mature predicted method called TIDE was applied to anticipate the immunotherapeutic effect of PD1 administration. We found a higher proportion of responders in the low-risk group (**Figure 8D**), and the lower TIDE score, indicating a higher response rate, verified the obtained finding. Moreover, the T-cell dysfunction score and cancer-associated fibroblasts were elevated in the high-risk group. According to previous reports, tumors with MSI tend to more easily respond to immunotherapy. The finding of a higher MSI score in the low-risk group supports the expectation (**Figure 8C**). We further compared the low-risk patients with rectal patients with MSI-H phenotype to investigate which group would achieve a better objective response from ICI treatment. Due to a small proportion of MSI-H patients in the TCGA dataset (4/157), we evaluated the MSI score for each patient with READ by the TIDE algorithm. The patients with MSI score higher than the median value were characterized as the MSI-H group, the others were classified as the MSI-L group. The result showed that there was no significant difference between the low-risk group and MSI-H group (*P* = 0.354, **Supplementary Figure S6**), indicating that the m5C regulator–based signature could be utilized as an addition to the current MSI classification, the combining of two methods to evaluate the responsiveness of ICI treatment will provide a novel perspective for precision medicine. We then performed a direct investigation by adopting an additional data set with the therapeutic information. We compared the survival rates of two subgroups by conducting Kaplan-Meier analysis, and found that the low-risk group had prolonged survival compared with the high-risk group despite an insignificant *P* value (*P* = 0.121, **Supplementary Figure S7**). The expression of immune checkpoints was higher in the low-risk group, which represents higher sensitivity toward ICI treatment (**Figure 8E**). Accordingly, the proportion of complete response/partial response (CR/PR) was remarkably higher in the low-risk group (**Figure 8F**), and the risk score was lower in the CR/PR subgroup (**Figure 8G**). Interestingly, compared with the immune-excluded high-risk group, the low-risk group revealed an immune inflammation phenotype (**Figure 8H**). These results solidly certified that the established signature had the ability to efficiently predict the immunotherapeutic efficacy for READ patients.

### The transcriptomic characteristics of the m^5^C methylation–based gene clusters

**Figure 9:**
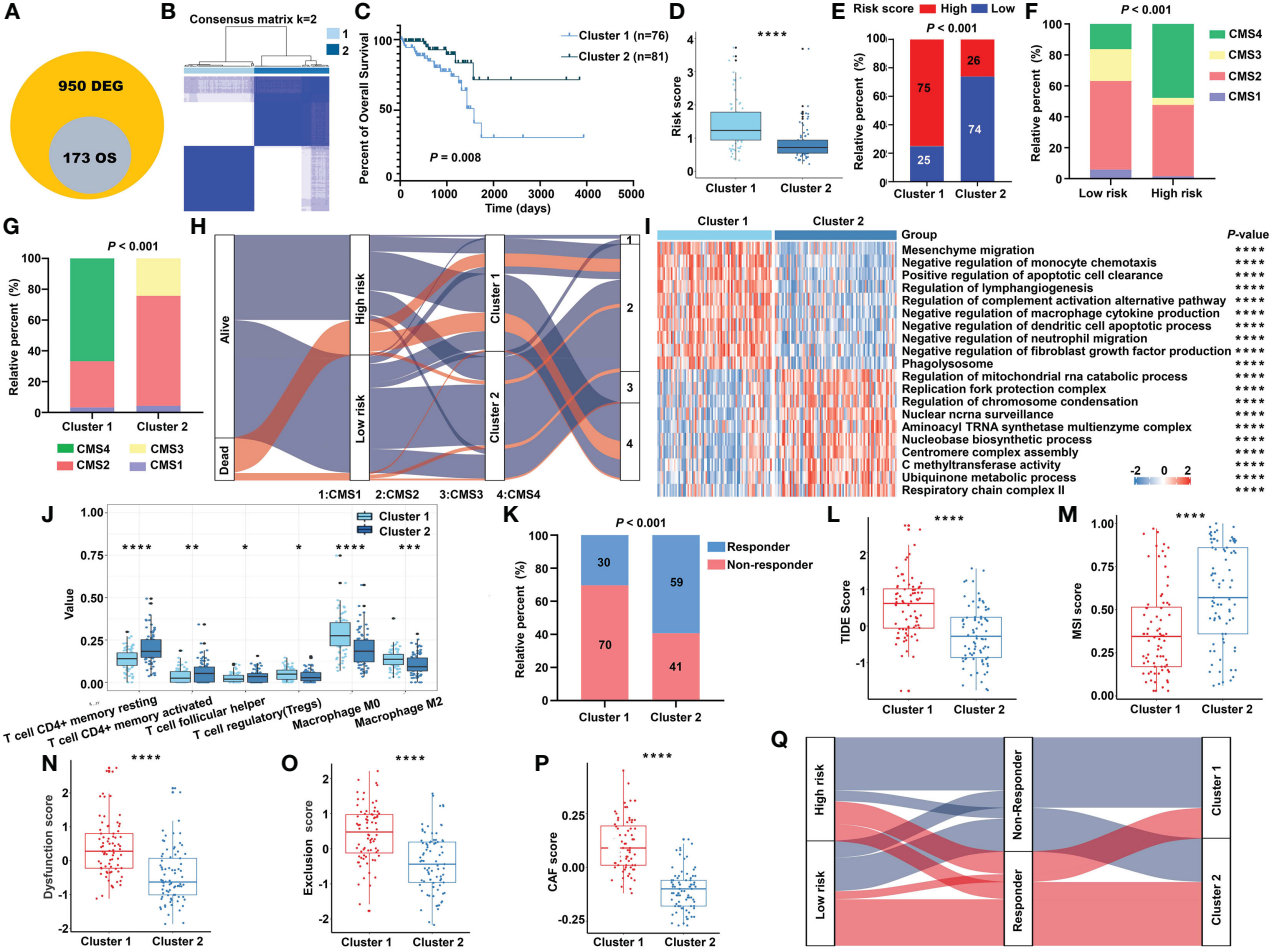
The transcriptomic characteristics of the m^5^C methylation–based gene clusters. **(A)** The intersection of DEGs and prognostic genes. **(B)** The unsupervised consensus cluster of the identified 173 genes. **(C)** The Kaplan–Meier survival curve for two clusters in the TCGA cohort. **(D)** The m^5^C signature–based risk score distribution between two clusters. **(E)** The proportion of READ patients with different risk status in cluster 1 and cluster 2 from the TCGA cohort. The CMS distribution among the risk groups **(F)** and clusters **(G)** separately. **(H)** Sankey diagram depicting the relationship of survival status, risk groups, clusters, and CMS classification. **(I)** The functional enrichment analysis on GO terms of the two clusters. **(J)** The immune cells infiltration between different clusters. **(K)** The proportion of responsive patients in the two clusters based on TIDE prediction. The distribution of TIDE score **(L)**, MSI score **(M)**, T-cell dysfunction score **(N)**, T-cell exclusion score **(O)**, and abundance of CAF **(P)** in cluster 1 and cluster 2. **(Q)** Sankey diagram connecting the two classification systems with the immune response. ****P < 0.0001; ***P < 0.001; **P < 0.01; *P < 0.05.

To further investigate the heterogeneity of different m^5^C RNA methylation regulator patterns, we identified 950 DEGs between the high-risk and low-risk groups. Subsequently, univariate Cox regression analysis was conducted to certify the genes with prognostic value, and finally, a total of 173 m^5^C RNA methylation regulator risk model–related genes were identified (**Figure 9A**). Unsupervised clustering analysis based on the expression of these 173 genes separated READ patients into two clusters, which we referred to as m^5^C RNA methylation gene clusters (**Figure 9B**). Survival analysis indicated that cluster 2 had a better prognosis (**Figure 9C**). Moreover, we found that cluster 1 had a higher risk score than that in cluster 2 (**Figure 9D**), and chi-squared tests also revealed a significant difference between the two clusters (**Figure 9E**). CMS stratification is considered a robust classification system and is currently used for CRC with distinguished features; among the four CMS subtypes, CMS4 mesenchymal tumors display worse overall survival and relapse-free survival (24). To evaluate the CMS status in different m^5^C regulator–based subgroups, we further compared the proportion of the CMS phenotypes by chi-squared tests. The high-risk group and cluster 1 category displayed a higher proportion of CMS4 compared with other categories (**Figures 9F, G**).

In line with the previous findings, cluster 1 was enriched mainly in cancer and immune system–related pathways, while process related to the functions of RNA methylation played an important role in cluster 2 (**Figure 9I**). Patients of cluster 2 had higher abundance of CD4+ T cells and helper T cells, while cluster 1 exhibited higher amount of immune-inhibiting cells, such as Tregs and macrophages (**Figure 9J**). The relationship of the survival status, m^5^C regulator–based risk model, m^5^C regulator gene clusters, and CMS phenotypes is summarized in a Sankey diagram (**Figure 9H**). The TIDE algorithm was carried out to predict the immunotherapeutic response relating with the clustering system. Accordingly, there were more responders in cluster 2 (**Figure 9K**), and the index integrating the lower TIDE score, higher MSI score, lower extent of T-cell dysfunction and exclusion, and lower abundance of cancer-associated fibroblast (CAF) consistently indicated a better responsive rate for patients of cluster 2 (**Figures 9L–P**). To make the outline clear, a Sankey diagram connecting with both the risk classification and clustering system was depicted (**Figure 9Q**). Above all, these results reinforced the notion that there were indeed two different m^5^C regulator–based groups in READ, which represented different clinical and immune features.

### Validation of the m^5^C methylation–based signature by TMA in patients with rectal cancer

To demonstrate the robustness and repeatability of the prognostic value of the established model, different laboratory assays were adopted. RT-qPCR was conducted to detect the mRNA expression of the signature’s factors in 26 pairs of rectal cancer tissues and corresponding normal tissues. The results showed that NSUN4 was highly expressed in normal tissue (**Figure S8A**). Examination of the correlation between the risk score and clinical parameters revealed a higher proportion of patients with no lymph node metastasis in the low-risk group compared with the high-risk group (**Figure S8B**).

**Figure 10:**
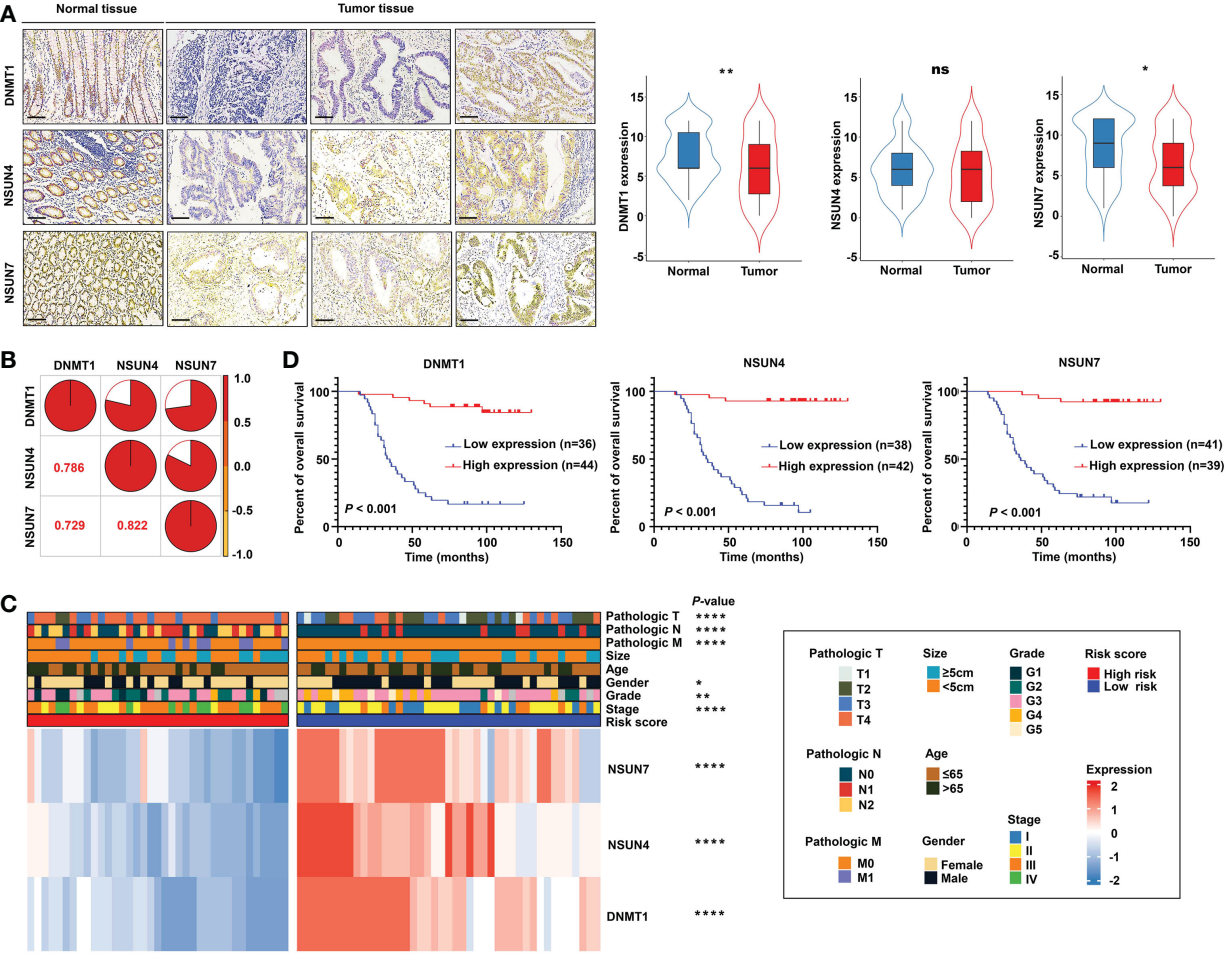
Validation of the m^5^C methylation– based signature by rectal cancer tissue microarray (TMA). **(A)** The differential expression of NSUN4, NSUN7, and DNMT1 between normal and tumor tissue; the representative micrographs show NSUN4, NSUN7, and DNMT1 IHC staining of 80 pairs of rectal cancer and corresponding normal rectal tissue samples in the rectal cancer TMA. **(B)** The correlation among the expression levels of NSUN4, NSUN7, and DNMT1. **(C)** The heat map depicting the association of the risk score, gene expression, and clinicopathological parameters. **(D)** Kaplan–Meier curves of differential NSUN4, NSUN7, and DNMT1 expression in the TMA cohort of rectal cancer. ****P < 0.0001; **P < 0.01; *P < 0.05; ns, not significant.

Next, we detected the protein expression levels of NSUN4, NSUN7, and DNMT1 *via* IHC staining in a tissue microarray containing 80 paired normal and tumor tissues. The clinical features of tissue microarray as the validation cohort are displayed in **Table S2**. The protein expression levels of the three m5C regulatory genes were analyzed using IHC staining, substantiating the findings obtained using the TCGA-READ dataset. The following analyses were based on the protein expression levels detected *via* IHC. The results revealed significant elevation of NSUN7 and DNMT1 in normal tissues compared with tumor tissues, while NSUN4 showed no obvious difference between the two groups (**Figure 10A**). Next, we investigated the relationship between the three genes using the Pearson correlation analysis. High correlation coefficients (> 0.7) shown in the correlation plot indicate that the protein expression levels of the three genes were closely associated (**Figure 10B**).

**Figure 11:**
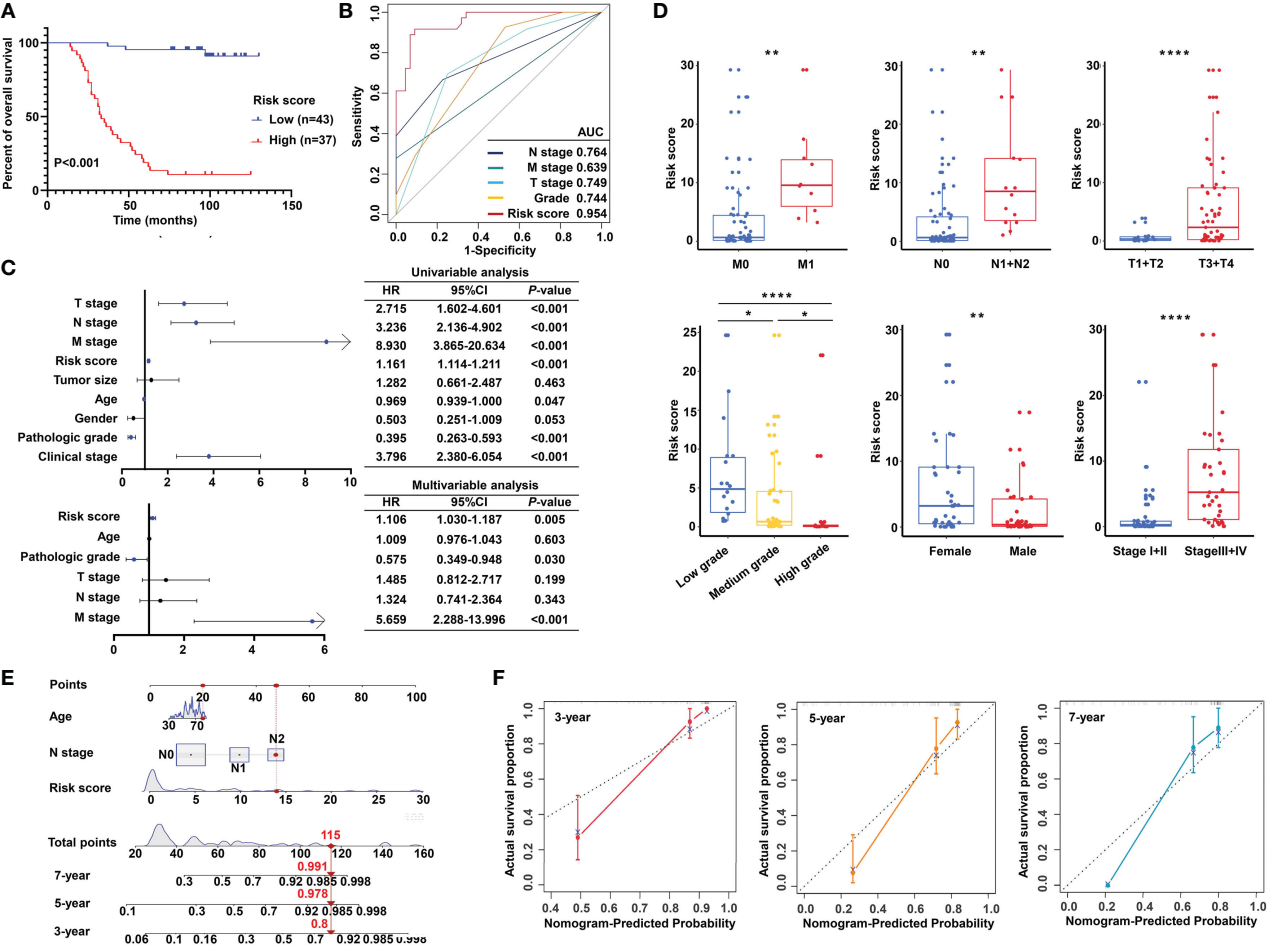
Validation of the m^5^C methylation–based signature by tissue microarray (TMA). **(A)** Kaplan–Meier survival curves show overall survival for low- and high-risk patients based on the rectal cancer TMA cohort. **(B)** The ROC curves depicted for the risk score and common clinical diagnostic indexes. **(C)** The univariate and multivariate Cox analysis of the risk score and clinicopathological indexes in the rectal cancer TMA patients. **(D)** The distribution of the risk score among various parameters including pathological TNM, stage, grade, and gender. **(E)** A nomogram integrated age, pathological N, and risk score was constructed for 3, 5, and 7 years based on the rectal cancer TMA cohort. **(F)** The calibration curves show the discrepancy between actual and nomogram-predicted survival probability in 3-, 5-, and 7-year nomograms. ****P < 0.0001; **P < 0.01; *P < 0.05.

Importantly, the KM survival curves demonstrated that the survival probability was significantly increased in the high expression group compared to the low expression group, according to the protein expression of an individual gene in the risk model (**Figure 10D**). We constructed a signature based on the protein expression of the three genes, in which the low-risk group showed prolonged survival compared with the high-risk group (**Figure 11A**). Remarkably, based on the IHC protein expression data, the risk score was correlated with clinical characteristics including pathologic TNM, gender, grade, and clinical stage (**Figure 10C**); this was further confirmed by a Wilcoxon test between the two subgroups (**Figure 11D**). To examine the significance of the established risk score, univariate and multivariate Cox regression analyses were conducted. Risk score, grade, and pathologic M remained independent factors after the above tests (**Figure 11C**). ROC analysis was exploited to inspect the superiority of the built risk score over other indexes (AUC of risk score = 0.954; AUC of grade = 0.744; AUC of pathologic N = 0.764; AUC of pathologic M = 0.639; AUC of pathologic T = 0.749; **Figure 11B**). To validate the efficiency of the nomogram generated based on the TCGA-READ dataset, we integrated the model factors, including risk score, age, and pathological N, to construct a nomogram based on the IHC independent cohort (**Figure 11E**). The C-index of the nomogram was 0.840, indicating a stable and robust predictive power. The subsequent calibration plots also revealed high concordance between the predicted probability of three-, five-, and seven-year OS and actual OS (**Figure 11F**). These results reinforced that our classification based on the m^5^C methylation regulators was potent and reliable in terms of prognostic significance for patients with rectal cancer.

In addition, the regulated genes associated with NSUN4, NSUN7 and DNMT1 using the STRING database were analyzed. Mitochondrial transcription termination factor 4 [MTERFD2], NOP14 nucleolar protein [NOP14] and RB transcriptional corepressor 1 [RB1] were identified to be closely related to NSUN4, NSUN7 and DNMT1 respectively with the highest predicted scores. As shown in **Supplementary Figure S9A**, NOP14 was significantly upregulated in rectal cancer tissues compared with normal tissues, while both MTERFD2 and RB1 showed no differences. Consistent with our results in TCGA, the immunohistochemistry results of the HPA database presented that the protein expression level of NOP14 was elevated in the tumor cells compared with the corresponding glandular cells, and mainly localized to the cytoplasmic and membranous nuclear (**Supplementary Figure S9B**). However, the protein expression of MTERFD2 or RB1 exhibited no difference between cancer tissues and normal tissues (**Supplementary Figure S9B**).

### Estimation of drug sensitivity for the m^5^C methylation–based signature

Based on the potential role played by the established m^5^C regulator signature in modulating the immunotherapies, we further investigated its clinical usefulness by measuring the IC50 value of different oncology drugs. According to the predictive model, we found that the effects of 10 commonly used drugs for READ were different between the two subgroups. Chemotherapeutic drugs, including camptothecin, 5-fluorouracil, cisplatin, oxaliplatin, and irinotecan, had a lower IC50 in the low-risk group.

**Figure 12:**
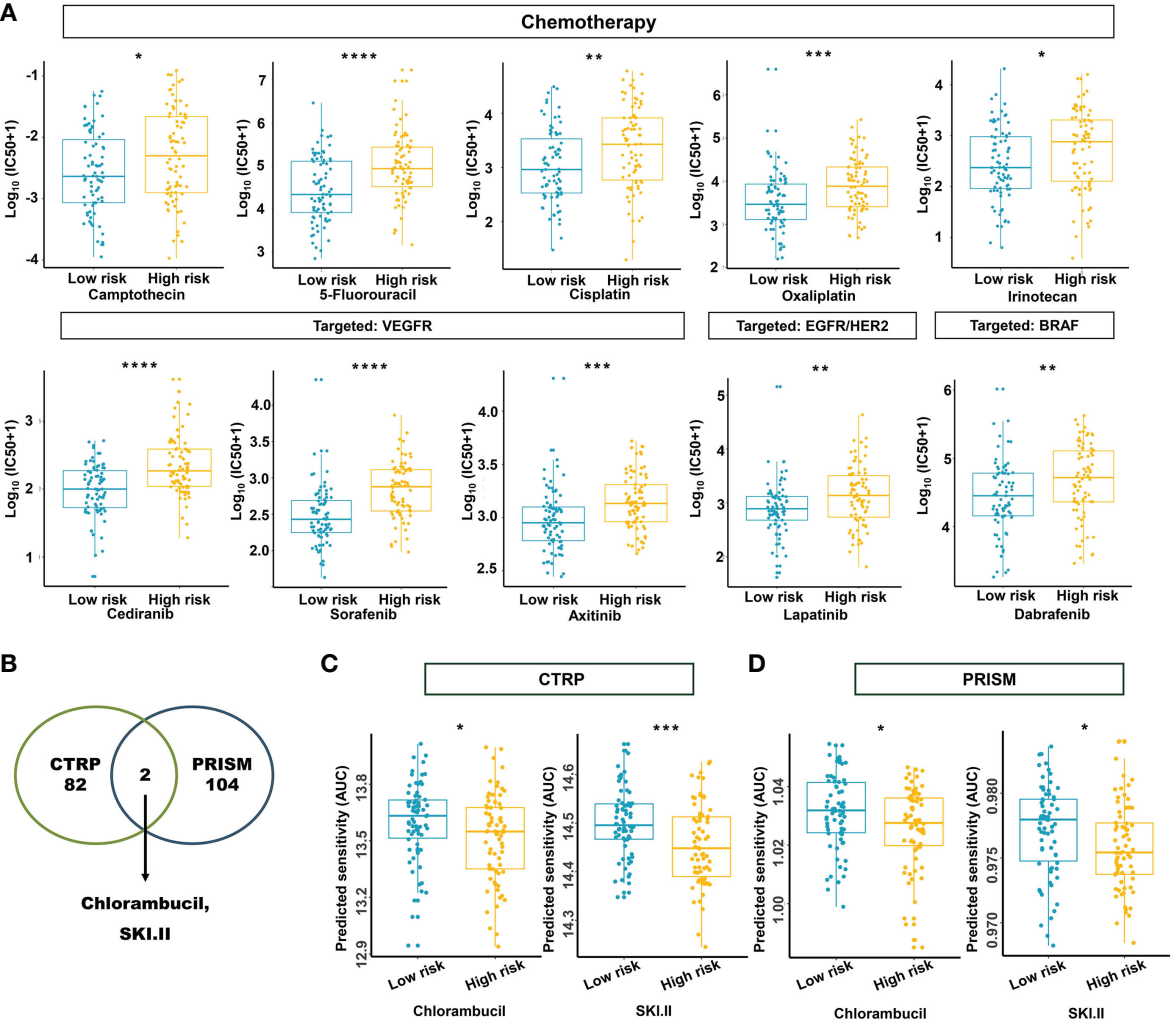
Estimation of drug sensitivity for the m^5^C methylation-based signature. **(A)** The evaluation of drug sensitivity including chemotherapeutics and small molecular drugs targeting VEGFR, EGFR/HER2, and BRAF. **(B)** Intersection of the identified drugs targeting high-risk patients between CTRP and PRISM databases. **(C, D)** The differential drug response analysis of CTRP- and PRISM-derived compounds targeting the high-risk group. IC50: half-maximal inhibitory concentration. ****P < 0.0001; ***P < 0.001; **P < 0.01; *P < 0.05.

Similarly, cediranib, sorafenib, and axitinib, which belong to VEGFR-targeted angiogenesis drugs, exhibited a lower IC50 in the low-risk group. EGFR/HER2 inhibitor lapatinib and BRAF inhibitor dabrafenib also followed that pattern (**Figure 12A**). To benefit high-risk patients, we further excavated both the CTRP and PRISM databases; two drugs specific to high-risk patients were found effective by intersecting the two sources (**Figures 12B–D**) and include chlorambucil and SKI.II. These results implied that our model could predict certain drug sensitivity that would be beneficial to different groups of READ patients.

## Discussion

Accumulating studies have revealed that colon and rectal cancer have distinct metastatic patterns, spread ratio, and drug response in patients (46). In multiple trials, individuals with rectal or colon cancer who received bevacizumab-containing regimens have shown different survival rates (47–49). In order to systematically distinguish colon and rectal cancer, Liang et al. even profiled specific biomarker and identified a key factor to tailor the medical treatment of patients with colon and rectal cancer (50). Available evidence indicates that colon and rectal cancer should be regarded as two specific cancers when considering clinical treatment. Therefore, we evaluated the prognostic significance of m^5^C regulators in COAD and READ separately. The results indicate that m^5^C might exert more impact on the prognosis of READ patients than COAD patients, which could be explained by the fact that colon and rectal cancer exhibit remarkably different genetic and epigenetic characteristics. A study enrolling 1,443 stage I–IV CRC patients revealed that the prevalence of MSI-high, BRAF mutations, and CIMP-high tumors rapidly decreased from the proximal colon to the rectum (51). Moreover, proximal tumors were more frequently MSI, hypermutated, BRAF mutant, and densely infiltrated by TIL, whereas distal tumors were CIN, HER1, and HER2 amplified, with active EGFR signaling and mostly non-BRAF-like characteristics according to an analysis of molecular features along anatomical sites in colon carcinomas of patients enrolled in the Pan European Trial Adjuvant Colon Cancer-3 (PETACC3) chemotherapy trial (52), indicating a great heterogeneity within CRC. Overall, the observation of significant difference between two types of cancer led us to focus our research on patients with rectal cancer.

RNA epigenetic modification is a crucial biological process. There is increasing evidence that the malfunction of RNA epigenetic modification leads to the deterioration of cancers. For example, NUSN4 has been found to affect the expression of mitochondrial DNA, which leads to a cascade of changes relating with the regulation of mammalian oxidative phosphorylation, finally resulting in the progression of cancers (53–55). The dysfunction of NSUN7 has been reported to result in male infertility (56), and NSUN7 is downregulated in prostate cancer compared with normal prostate tissue, acting as a protective factor in patients with prostate cancer (57). Additionally, DNMT1 is an important methyltransferase for the stable process of RNA methylation. It is associated with a series of cancers, including breast cancer, thyroid cancer, pancreatic cancer, and hepatocellular carcinoma (58–61). Here, based on the gene expression of m^5^C regulators (NSUN4, NSUN7, DNMT1), we established a signature that could effectively distinguish the prognosis of READ patients. A weak positive correlation was found between the three genes based on the TCGA-READ, indicating the independence of the three genes in the current model, and their cumulative effect can endow the model biological significance at the mRNA expression level. The constructed signature, age, and pathologic N act as independent prognostic factors in rectal cancer. Moreover, the signature could predict risk for patients of different age groups and N stages. Notably, the signature failed to distinguish the survival status of patients in the N1/N2 stage. At the advanced stage of the disease, colorectal cancer–associated immune infiltrates can be highly heterogeneous and can vary their phenotypes in a spatiotemporal manner (62, 63). Moreover, various factors such as intestinal obstruction, gastro-intestinal bleeding, malnutrition, liver metastasis, and other maladies can cause death in advanced colorectal cancer. All the above uncertainties could account for the reason that risk score only exhibited a trend (*P* = 0.089) when stratifying the overall survival of patients in the pathological N1/N2 stage due to small sample size. Differentiating the survival status of N0 patients is significant for early intervention. Colorectal cancer develops asymptomatically, leading to the difficulty in diagnosis and thus progressing into the advanced stage, which requires considerable efforts to treat (64). The established risk score could efficiently evaluate the hazards of patients in the pathological N0 stage and predict patients who are at high risk of developing advanced stage cancer, and the emphasis placed on these patients will benefit them clinically. Since IHC enables a pathologist to examine gene expression at the protein level within the context of histologically interpretable tissue sections, it is a reliable method for confirming expression signatures discovered by RNA sequencing. Therefore, to further substantiate the results of the bioinformatics analysis, TMAs from patients with rectal cancer were immunohistochemically stained for NSUN4, NSUN7, and DNMT1. The stained slides were evaluated for calculating risk score. In concordance with the TCGA data mining, the risk score was able to differentiate the prognosis of patients with rectal cancer well and determine their survival as an independent prognostic factor, and the nomogram integrating risk score, age, and pathological N could serve as a reliable indicator in predicting the survival probability of patients with rectal cancer. Since IHC is carried out on commonly processed clinical tissue samples, validated IHC assays could be easily applied in clinical diagnostics. To facilitate the clinical use, we developed a nomogram with high accuracy and robustness. Our findings together suggest that the built signature based on m^5^C RNA regulators is highly involved in the progression of rectal cancer and could serve for effective risk stratification in patients with rectal cancer.

There is increasing evidence relating the m^5^C modification with innate immunity as well as antitumor effect through a complex crosstalk among various m^5^C regulators. We found that the established signature could effectively determine the TIME infiltration patterns. The interplay between tumor and immunity begins when tumor antigens are presented by dendritic cells and activate CD8+ T cells and CD4+ T cells to exert cytotoxic effects (65). Moreover, cancer cells can suppress immune system, leading to an inhibitory TIME to escape immune surveillance with the increase of Tregs and MDSC. As revealed in our analysis integrating CYBERSORT, TIMER, EPIC, and ssGSEA algorithms, the low-risk group was characterized by the activation of adaptive immunity, with the increasing abundance of CD4+ T cells, CD8+ T cells, B cells, and myeloid dendritic cells. The high-risk group was characterized by the suppression of immunity, accompanied by upregulation of Tregs and MDSC. The ratio analysis further explained that compared with the high-risk group, the scales of CD8+/CD4+ regulatory T cells and pro-/anti-inflammatory cytokines were higher in the low-risk group. According to different functions, macrophages could be classified into two categories: classically activated macrophages (M1), mainly acting as a tumor-killer role, and alternatively activated macrophages (M2), which function to promote tumor cells (66). As indicated in our results, the ratio of M1 to M2 macrophages was elevated in the low-risk group. m5C RNA methylation regulators have already demonstrated the efficacy for predicting prognosis and regulating TIME in various cancers (18, 67, 68), suggesting the potential value in pan-cancer analysis. Consistent with the current knowledge, our model showed a predictive accuracy in prognosis and in TIME cell infiltration characterization among READ patients.

The signatures derived from m6A/m5C/m1A RNA methylation regulators were widely explored in recent studies. Commonly, the signatures could characterize the immune landscape of cancer patients and further predict the efficacy of immunotherapy (69, 70). m6A modification is one of the most researched RNA methylation patterns. The “writer”, “reader”, and “eraser” of m6A modification correlated closely with immune infiltrating cells (71), giving rise to the application of m6A RNA methylation regulators in predicting immune efficacy. Two m6A RNA demethylases, FTO and ALKBH5 were targeted to develop inhibitors (72–74), providing insights into understanding the roles of m6A RNA methylation involved in multiple diseases. m5C RNA modification is regarded as a novel methylated process in eukaryotes. Small-molecular inhibitors targeting m5C RNA methylation regulators were conceived by proof-of-concept studies, while, specific m5C inhibitors have yet to be developed (75). m5C RNA methylation regulators can impact the process of tumorigenesis by regulating TIME in cancers, so that inspecting the roles involved in the immune system will give hints to personalized immunotherapy strategies making. m1A methylation modification is a new form of modification of RNA, thus, studies on m1A modification in tumorigenesis are rarely reported. Although several signatures based on m1A modification were built to guild effective immunotherapy strategies (70, 76), controversies remained when detecting the m1A methylation sites (77, 78). More efficient and accurate technologies need to be developed to uncover the m1A modification sites to fully exploit the value of m1A modification in anti-tumor immunotherapies. More effort is deserved to understand the complex network regulated by different kinds of RNA methylations in modulating tumor-immune interactions. However, in the current study, we focused on the prospects of m5C methylation regulator as the predictive biomarker for ICIs treatment.

The quantity of cancer mutations is reflected by TMB. Major histocompatibility complex proteins turn mutations into neoantigens and further present them to T cells. More neoantigens are produced by higher TMB, which in turn boosts the likelihood that T cells recognition will happen, clinically corresponding with improved ICI outcomes (79). Several studies have shown that high TMB and neoantigens correlated with better prognosis in non-small-cell lung cancer (NSCLC) and melanoma (80–82). In this study, the low-risk group possessed more mutations and higher level of neoantigens than the high-risk group, suggesting a better response to immunotherapy within the low-risk group. We also identified the stratifying efficiency of the model in patients with same status of neoantigens and TMB. The prognostic power of the established model was superior to neoantigens or TMB. These results indicated that our model had the potential to combine with or modify existing biomarkers, achieving improved accuracy in prognostic prediction. In addition to using neoantigens and TMB, immune checkpoints can be inhibited to enable T cell functions. By allowing T-cell reactivation, ICIs have revolutionized cancer treatment (83). The Food and Drug Administration (FDA) has approved six inhibitors of the programmed cell death protein pathway (PD1/PD-L1) and an inhibitor of the CTLA-4 for use in treating various cancers (84–86). In our study, we observed a weak correlation between model factors and immune checkpoints except for NSUN4. In fact, immune checkpoints alone are not sufficient to predict the efficiency of the immunotherapy due to a highly complex immune tumor microenvironment, which could be generalized by a cancer immunity cycle (87). Several studies have suggested integrating multiple biomarkers to predict the immune response, including tumor-infiltrating lymphocytes, mutational burden, immune gene signatures, and multiplex immunohistochemistry (88, 89). TIDE is a reliable surrogate biomarker that could accurately predict immune checkpoint blockade (ICB) response by measuring the tumor immune escape, and it even performed better than PD-L1 expression in melanoma; that is, a higher TIDE score is associated with worse ICB response and worse patient survival under anti-PD1 and anti-CTLA4 therapies (90). According to our previous studies and others, the immune landscape is crucial in assessing the efficacy of immunotherapy and chemotherapy targeting CRC patients (91–94). However, the role of m^5^C RNA methylation regulators in patients with rectal cancer is still unclear. In the current research, we found that responders were proportionally more frequent in the low-risk group compared with the high-risk group. The lower TIDE prediction score, T-cell dysfunction score, CAF, and higher MSI score in the low-risk group indicate a good function of T cells with high infiltration by cytotoxic T lymphocyte (CTL), further explaining why the low-risk group was more sensitive to immunotherapy. In addition, in IMvigor210 cohort with the determined immune response, these results were well confirmed. Besides, drug sensitivity was examined between the low- and high-risk groups by performing the R package “oncoPredict”. Apparently, the majority of the chemotherapeutic agents achieved their efficacy among the low-risk group; nonetheless, drugs targeting specifically the high-risk group were also investigated by screening drugs of CTRP and PRISM databases. The AUC values between two risk groups were compared and drugs intended to the high-risk group were selected. Finally, chlorambucil and SKI.II were found in both the CTRP and PRISM databases. These results indicated the built risk model was a trustworthy and robust approach for a thorough evaluation of each patient’s therapeutic response, which could benefit the precision treatment combining immunotherapy and chemotherapy for patients with rectal cancer.

Furthermore, the mRNA transcriptome differences between the high- and low-risk groups have been investigated. They were highly involved in the cancer and immune system–related biological pathways. The DEGs with prognostic efficacy were considered m^5^C-related signature genes. Two genomic subgroups were discovered based on the m^5^C signature genes, which could significantly predict the survival and immune response of READ patients, and were substantially connected with immunological activity. These results were similar to the stratification of the risk model. This once again showed the power of the m^5^C regulator–based signature in shaping the landscapes of the READ patients. Thus, a thorough analysis of m^5^C alteration patterns will definitely improve the precision classification and therapeutic strategy for patients with READ.

Despite the encouraging findings, the current study included several limitations. First, the gathered data were analyzed retrospectively, and multicenter research and large-scale prospective investigation are required to confirm and rectify our model. Second, the specific crosstalk between these m^5^C methylation regulators and corresponding immune characteristics remains unrevealed. The regulatory network of the three genes in rectal cancer needs to be further investigated. As for now, the genes regulated by NSUN4 and NSUN7 still need to be identified. Research related to the regulatory role of the three genes could provide novel insights into the mechanisms of the built signature. Third, the ability of this signature to predict immunotherapeutic or chemotherapeutic response was assessed indirectly due to the lack of data from patients with rectal cancer receiving related treatments. Research focusing on the therapeutic effect of the current signatures should be done *in vitro* and *in vivo* in the future. Fourth, the sizes of clinical tissue specimens for TMA and RT-qPCR assay used in our independent validation cohorts were limited, and more samples are expected to verify the m5C methylation regulator –based signature in the future.

In conclusion, the established risk model could be used to comprehensively evaluate the prognosis and the clinical response to adjuvant chemotherapy and immunotherapy among patients with rectal cancer. Moreover, the complex characteristic of the TIME cell infiltration could be effectively illustrated by the built signatures based on m^5^C regulators, producing a number of novel insights for cancer immunotherapy. Our research offers fresh approaches for predicting survival status, enhancing immunotherapy outcomes, disclosing various tumor immune phenotypes, and conclusively, advancing tailored cancer treatment in the future.

## Data availability statement

The original contributions presented in the study are included in the article/**Supplementary Material**. Further inquiries can be directed to the corresponding author.

## Ethics statement

The studies involving human participants were reviewed and approved by the Research Ethics Committee of The Affiliated Hospital of Qingdao University. The patients/participants provided their written informed consent to participate in this study.

## Author contributions

MY and RZ conceived and designed the experiments. MY supervised the work. JZ, RZ, MZ, and ZY provisioned the study materials or patients. RZ, JZ, TL, and SL collected and assembled the data. RZ, ZZ, WW, and FZ analyzed and interpreted the data. MY and RZ wrote the article. The final manuscript was read and approved by all authors. All authors contributed to the article and approved the submitted version.
