# Supplementary material for: Developing an m5C regulator–mediated RNA methylation modification signature to predict prognosis and immunotherapy efficacy in rectal cancer
Source: Front Immunol. 2023 Feb 22;14:1054700. doi: 10.3389/fimmu.2023.1054700 (PMC9992543; doi:10.3389/fimmu.2023.1054700)
Supplement: Supplementary file 1 [file DataSheet_1.docx]

**SUPPLEMENTARY MATERIALS**


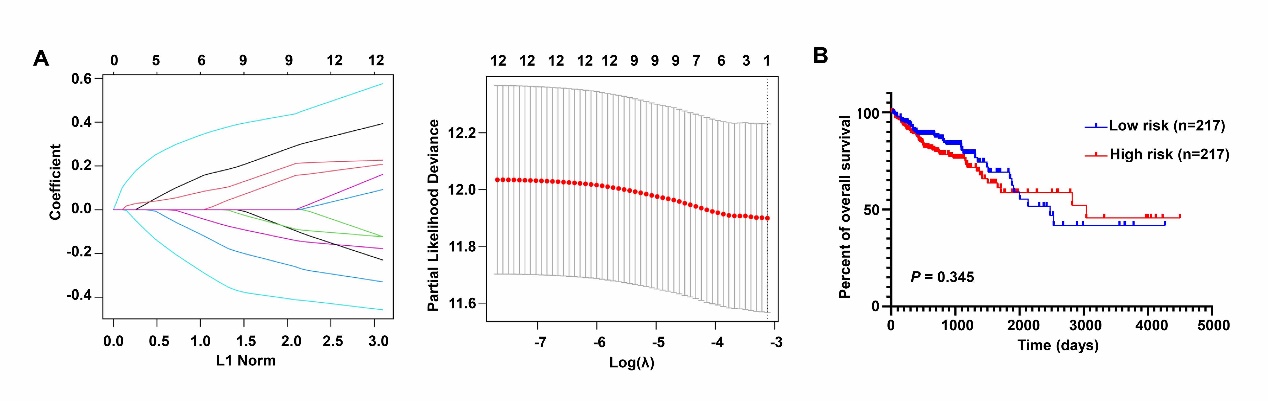


**Supplementary Figure S1 (A)** The LASSO processes for m^5^C methylation regulators based on the COAD patients in TCGA. **(B)** Kaplan–Meier curve of the high- and low-risk groups separated by the three-gene–based signature in TCGA-COAD patients.


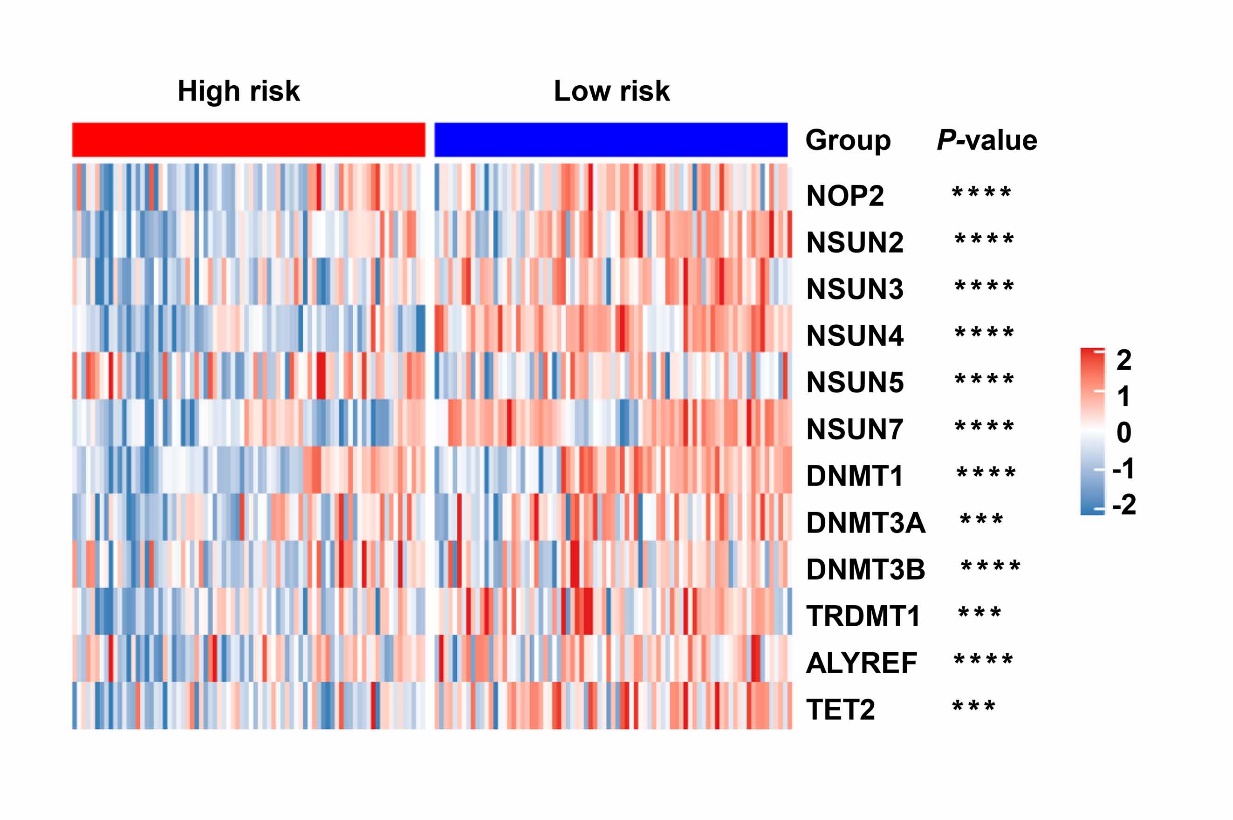
**Supplementary Figure S2** Heat map integrating the expression of m^5^C methylation regulators in the high- and low-risk groups based on the TCGA cohort.

**
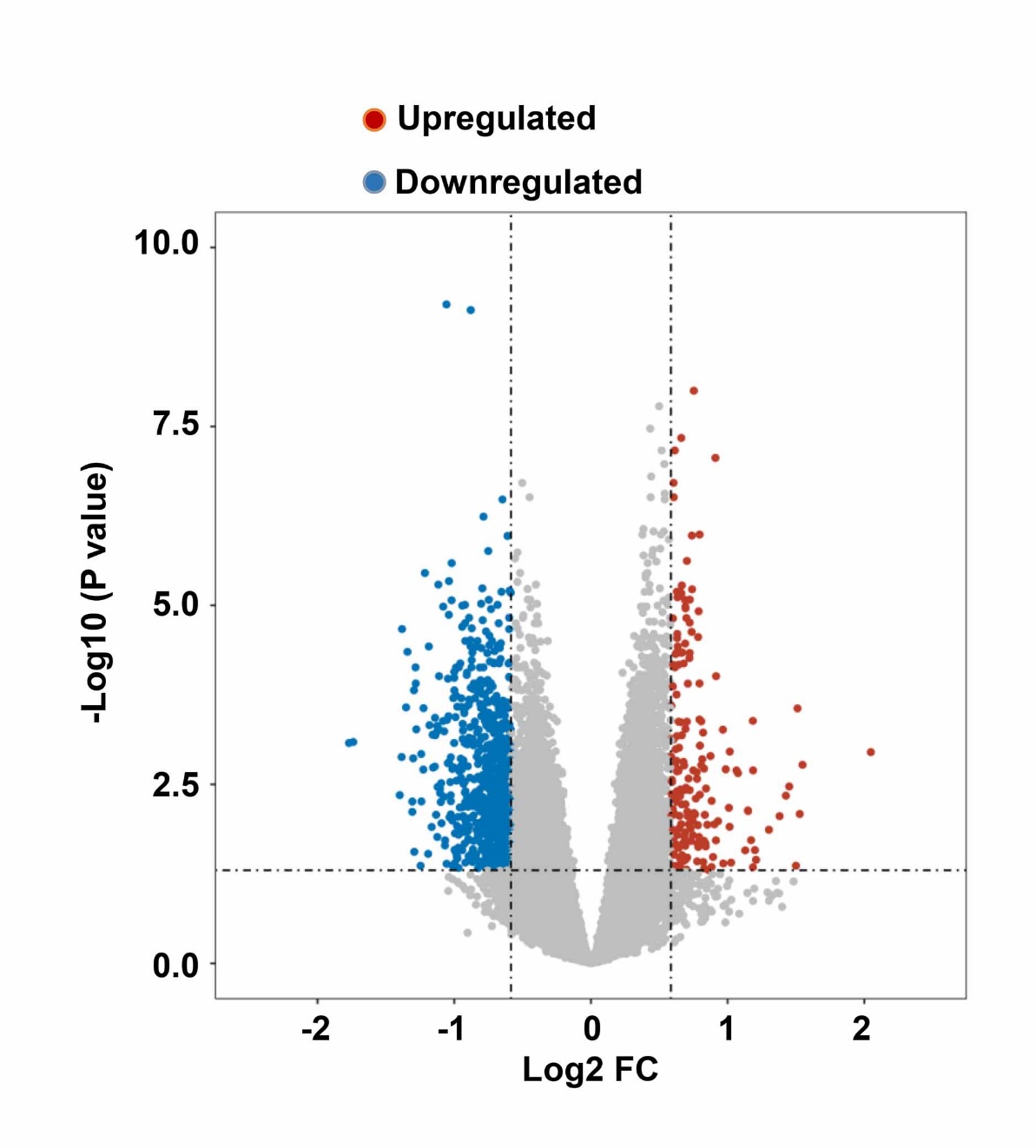
**

**Supplementary Figure S3** The volcano plot for DEGs between the high- and low-risk subgroups. *****P* < 0.0001; ****P* < 0.001; ***P* < 0.01; **P* < 0.05.

**
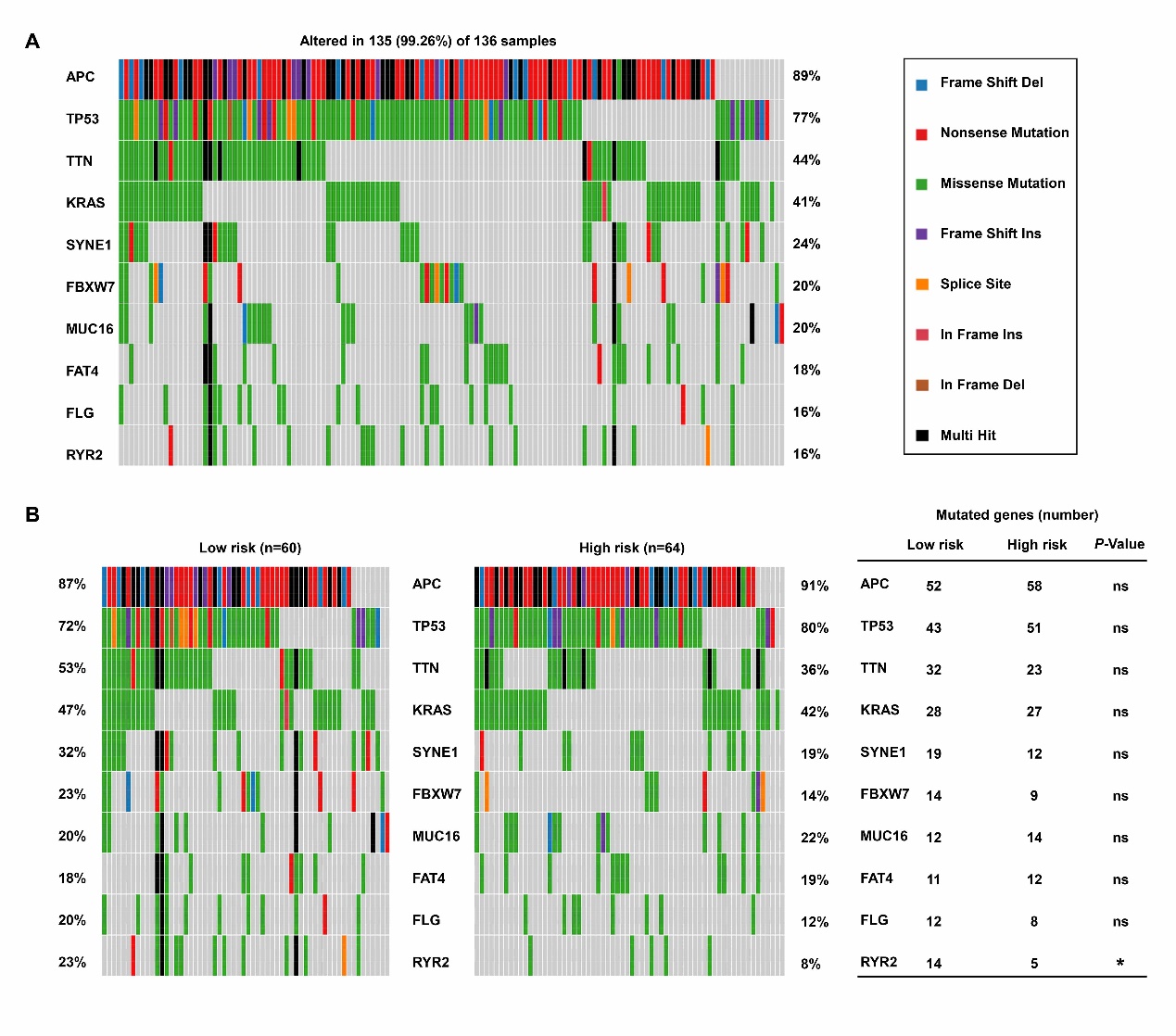
**

**Supplementary Figure S4 (A)** Waterfall plot of mutation frequencies of top 10 genes in TCGA-READ patients, with various color annotations to represent different mutation types. **(B)** Waterfall plot indicating the differences in the top 10 mutation frequencies of genes in TCGA-READ patients of the low- and high- risk groups. *****P* < 0.0001; ****P* < 0.001; ***P* < 0.01; **P* < 0.05.


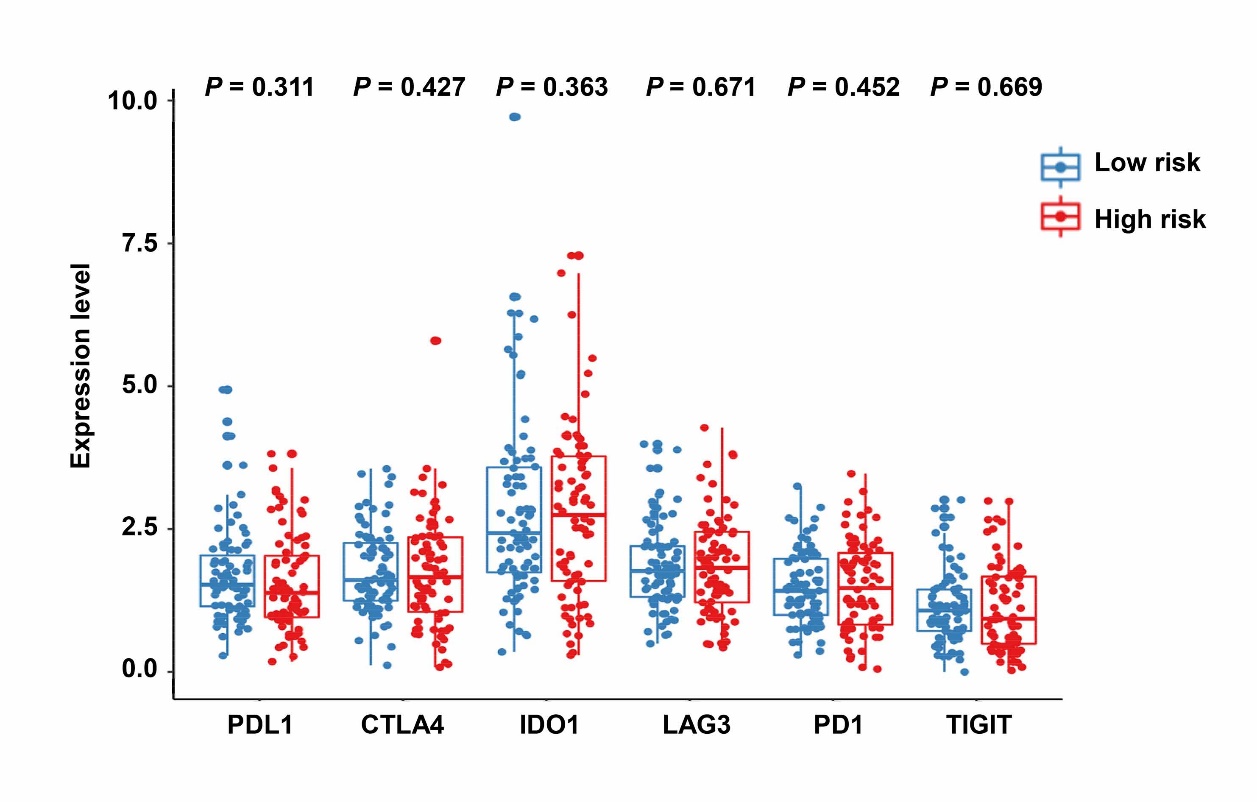


**Supplementary Figure S5** The differential analysis for immune checkpoints between the low- and high- risk groups based on TCGA-READ dataset.


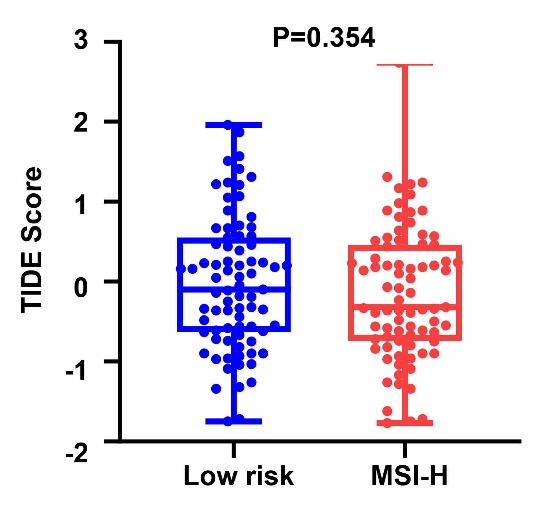


***P* = 0.354**

**Supplementary Figure S6** The distribution of TIDE score in low-risk and MSI-H groups from patients with READ based on the TCGA dataset.


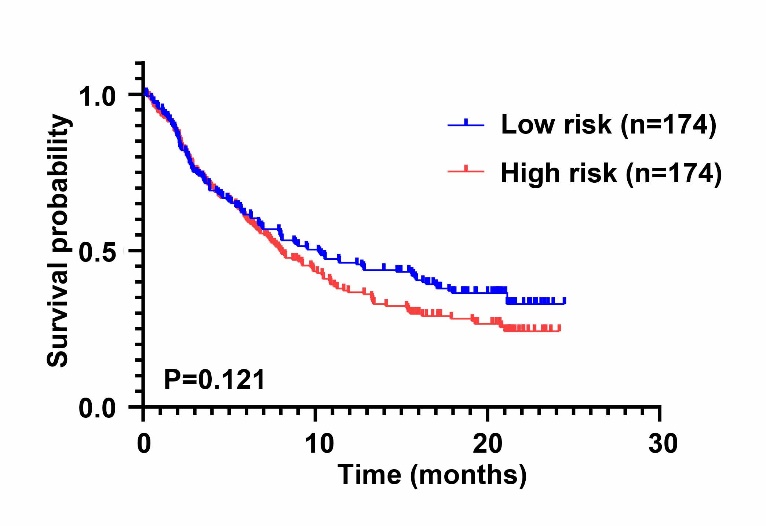


***P* = 0.121**

**Supplementary Figure S7** The Kaplan-Meier survival curve for low- and high-risk patients in the IMvigor210 cohort.


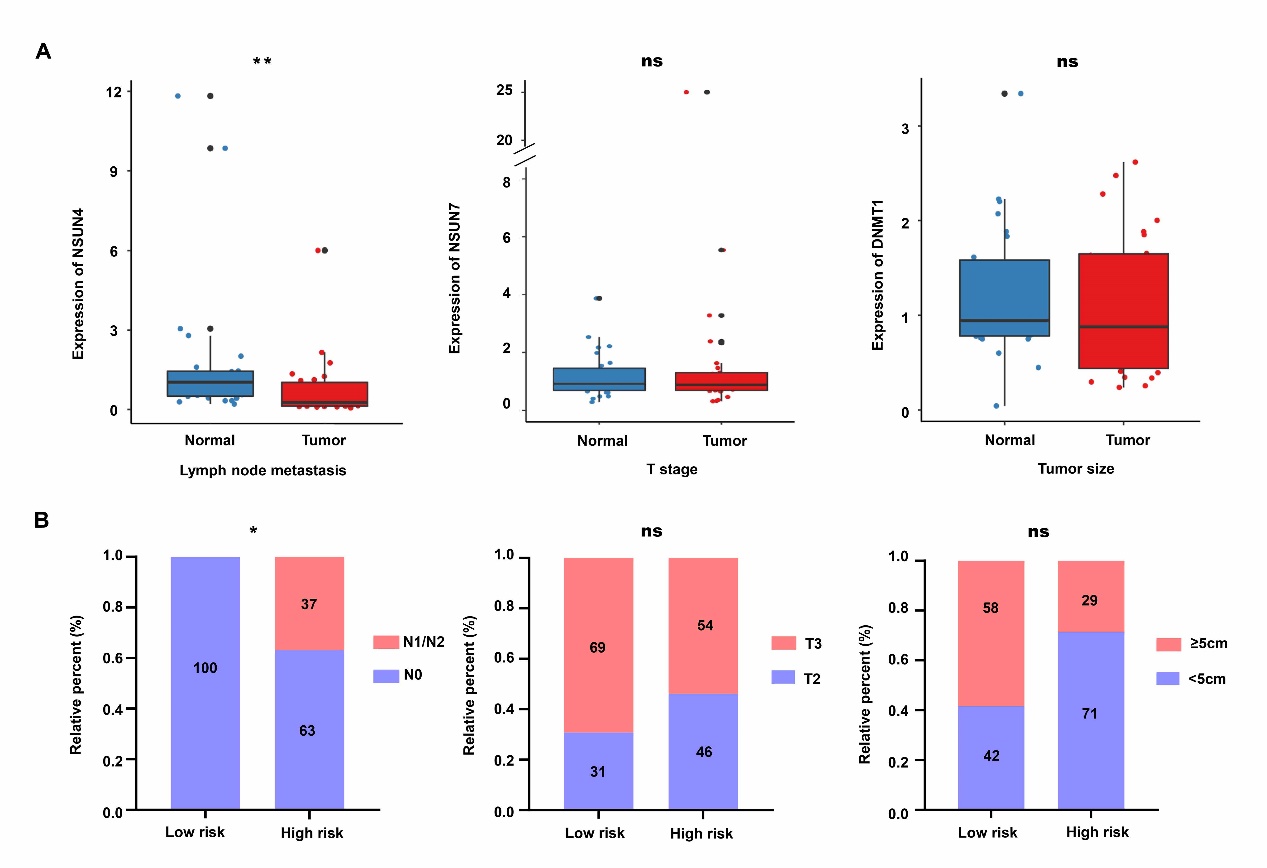


**Supplementary Figure S8 (A)** The mRNA expression of NSUN4, NSUN7, and DNMT1 in 26 pairs of READ and corresponding normal tissues. **(B)** The histograms display the correlation of the m^5^C methylation regulator–based signature and clinicopathological characteristics in READ patients. *****P* < 0.0001; ****P* < 0.001; ***P* < 0.01; **P* < 0.05; ns, not significant.


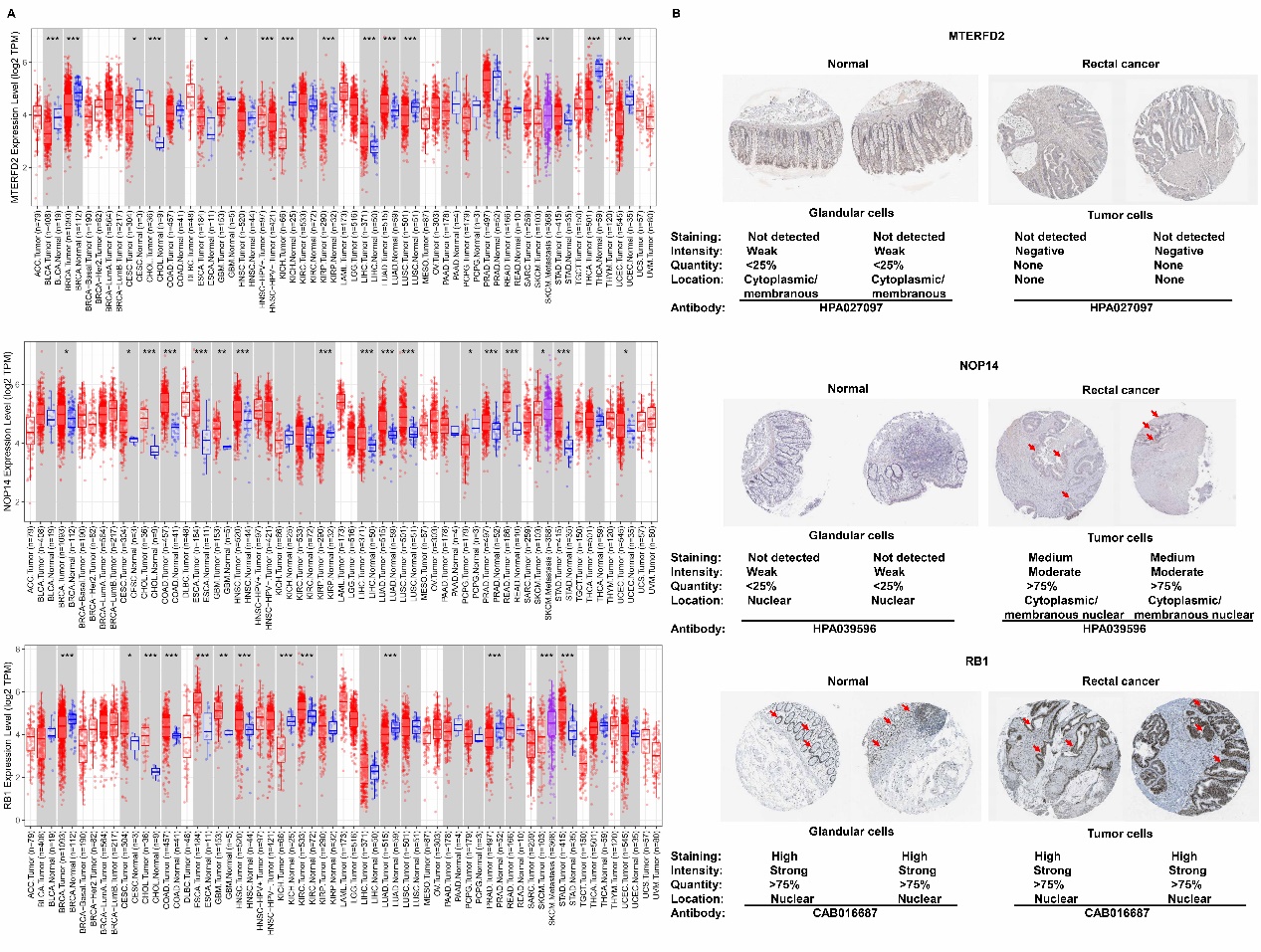


**Supplementary Figure S9** Differences in the expression of MTERFD2, NOP14 and RB1 in normal tissues and tumor tissues. **(A)** The differential expression of MTERFD2, NOP14 and RB1 between tumor and corresponding normal tissues from different cancer types based on the TIMER website. **(B)** Protein expression map of MTERFD2, NOP14 and RB1 in the HPA database.


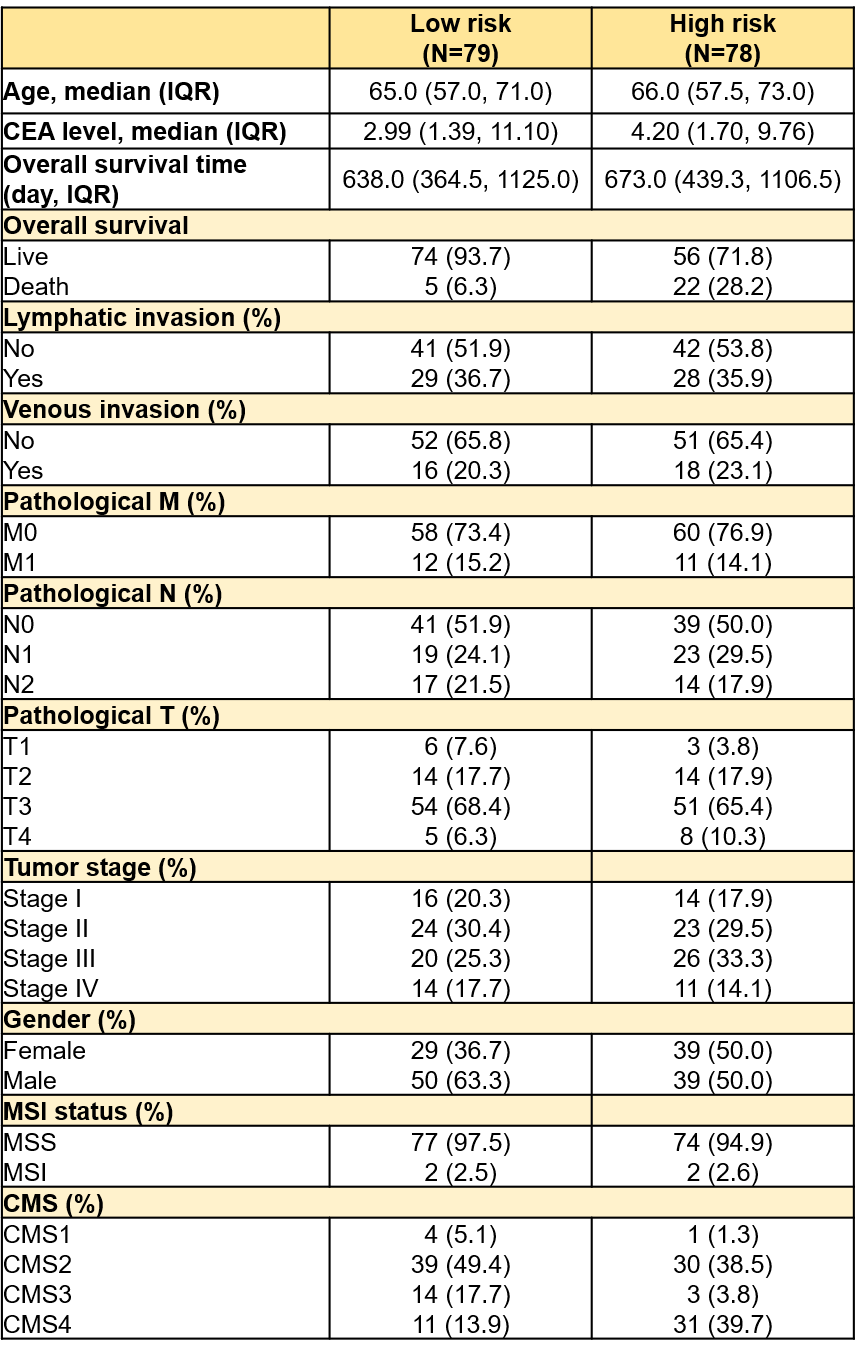


**Supplementary Table S1 Clinical characteristics of the patients with READ in TCGA cohort**

**Abbreviations: IQR, interquartile range**

**Description**


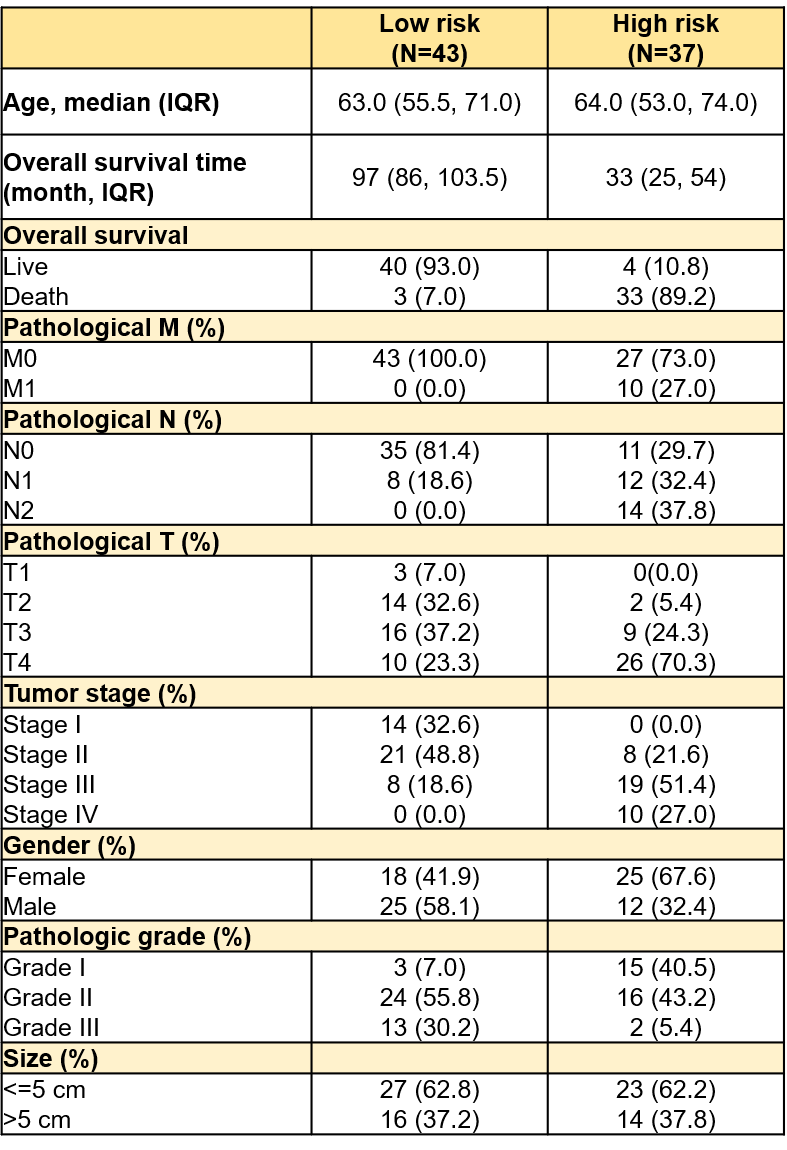


**Supplementary Table S2 Clinical characteristics of the patients with rectal cancer in TMA-IHC cohort**

**Abbreviations: IQR, interquartile range**

**Description**
